# A multiphase and multiscale mechanistic model for hot air drying of *shiitake* mushroom

**DOI:** 10.1016/j.crfs.2025.101296

**Published:** 2025-12-29

**Authors:** Lina Hu, Xin Jin, Yitong Xie, Jinfeng Bi, Ruud G.M. van der Sman

**Affiliations:** a Institute of Food Science and Technology, Chinese Academy of Agricultural Sciences (CAAS) / Key Laboratory of Agro-Products Processing, Ministry of Agriculture and Rural Affairs, Beijing, 100193, PR China; b Food Process Engineering, Agrotechnology and Food Sciences Group, Wageningen University & Research, Bornse Weilanden 9, Wageningen, 6708 WG, the Netherlands; c Food and Biobased Research, Wageningen University & Research, Bornse Weilanden 9, Wageningen, 6708WG, the Netherlands

**Keywords:** Mechanistic model, Mushrooms, NMR, Cell membrane integrity

## Abstract

A mechanistic multiphase, multiscale model is proposed to simulate mushroom drying, incorporating shrinkage, Flory-Huggins theory for water activity, viscoelasticity, alongside the heat and mass transfer. The model describes moisture and temperature evolution, capturing unique features of mushroom drying dynamics, such as sustained internal evaporative cooling, and a rapid increase in product temperature near the end of drying. This model enables the interpretation of T_2_ NMR investigations of drying, thereby revealing the conditions for the loss of cell membrane integrity. By integrating conductivity and NMR data, critical thresholds for maintaining cell membrane integrity were identified. The findings support further optimization of serial drying processes, while preserving membrane integrity for optimal rehydration.

## Highlights

• A mechanistic model integrates heat/mass transfer with key material properties.
• It captures unique drying dynamics like evaporative cooling and final temperature spike.
• It enables NMR T_2_ interpretation, identifying critical cell membrane integrity thresholds.

## Graphical abstract

**Figure:**
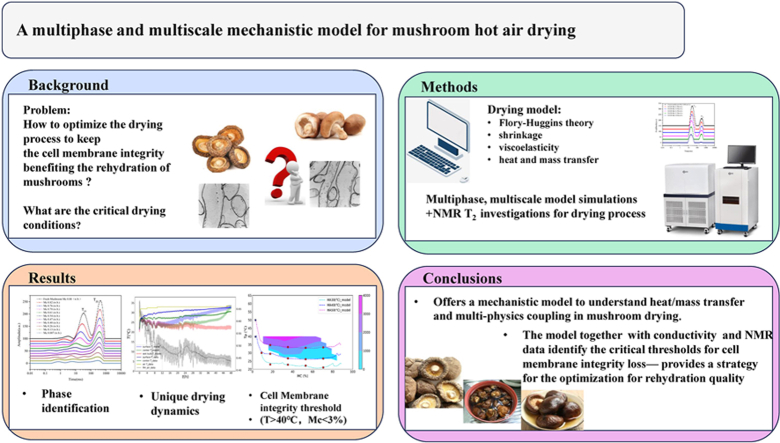

## 1 Introduction

Drying is a critical process for preserving the quality and extending the shelf life of *shiitake* mushrooms. However, it involves complex thermo-hydro-mechanical phenomena that affect final product properties, particularly the rehydration capacity. Currently, conventional optimization approaches of drying in the industry, particularly when adjusting factors such as air temperature, humidity, and velocity, are labor-intensive and energy-demanding. Mechanistic modeling offers an effective alternative, enabling detailed simulations of spatial temperature and moisture distributions. Such models provide insights that may be challenging to obtain directly from experiments (Castro et al., 2018). Compared with empirical methods, mechanistic modeling allows for the investigation of the individual roles of various transport mechanisms (Jiang et al., 2017). While numerous studies have focused on identifying empirical drying models that fit mushroom drying data (Dinani et al., 2014; Guo et al., 2014; Hou et al., 2024; Peter et al., 2021; Tran et al., 2020), few have systematically examined the underlying physical mechanisms. In contrast, mechanistic drying models have been well developed for other food materials, providing valuable insight into heat and mass transfer processes (Adduci et al., 2024; Castro et al., 2018; Datta et al., 2022; Datta, 2007a, 2007b; Halder et al., 2011; Hou et al., 2020). Although a multiphase porous media model with coupled thermo-hydromechanical phenomena has been proposed for mushroom drying (Zhu et al., 2021), it has not yet incorporated physical aspects, such as changes in cell membrane integrity and viscoelastic properties during drying.

In previous studies (Hu et al., 2021, 2022, 2023), it was demonstrated that cell membrane integrity, which is closely linked to temperature and moisture content, critically governs the rehydration quality of dried *shiitake* mushrooms. In drying models, the relationship between water activity and moisture content, which is typically represented by moisture sorption isotherms (MSIs), is critical. For cellular food materials, both viscoelastic relaxation and cell membrane integrity have been reported to influence moisture sorption behavior (Fredriksson and Thybring, 2018; Hill and Beck, 2017; Hill et al., 2012; Lee and Lee, 2008; Patera et al., 2016; Pedro et al., 2010; Salmén and Larsson, 2018). For mushrooms, it has been found that viscoelastic relaxation contributes to sorption hysteresis, while the cell membrane integrity affects the water-holding capacity at high relative humidity (Hu et al., 2023). Therefore, incorporating viscoelastic relaxation into drying models can enhance the understanding of the heat and mass transfer phenomena during mushroom drying. To understand the changes in cell membrane integrity during drying, it necessitates an accurate knowledge of temperature and moisture evolution, which can be predicted with the drying model and hence supports rational drying design and efficient optimization.

To predict moisture sorption isotherms, theoretical composition-based models, such as the Flory-Huggins Free Volume theory (FHFV), have been developed, which can then be integrated into drying models (Hu et al., 2023; Jin et al., 2014; Oliver and Meinders, 2011; van der Sman, 2013). In these models, the complex cellular structure of plant foods is approximated as two coexisting thermodynamic phases: one containing all biopolymers (cytoplasm and cell wall) and the other containing all solutes (vacuole), separated by a cell membrane. The membrane restricts solute transport but allows moisture exchange, with water partitioning determined by the local equilibrium (equal water activity in both phases) (Jin et al., 2014). Low-Field Nuclear Magnetic Resonance (LF-NMR) can identify distinct water populations within cells by measuring the transverse relaxation time (T_2_), as water molecules in different chemical environments exhibit different T_2_ values, with higher T_2_ values corresponding to more mobile water, and lower T_2_ values indicating less mobile water (Qiao et al., 2005; Xu et al., 2017; Zhang and McCarthy, 2012). The two thermodynamic phases in mushrooms may be identified by different peaks in the NMR relaxation time spectrum, potentially indicating the cell membrane integrity during drying.

The aim of this study is to develop a mechanistic model to examine mushroom drying processes, explicitly accounting for the complex heat and mass transfer phenomena within their porous structures. The model aims to be mechanistic, as much as possible, by incorporating: 1) a composition-based moisture sorption isotherm derived from the Flory-Huggins theory, considering cellular structure, stress effects, and viscoelastic relaxation; 2) a mechanistic description of moisture diffusion through bound water and the gas phase; and 3) thermal conductivity based on composition and porosity. Although ideally, deformation and stresses can be modeled by solving momentum balances, current theories for the large deformation of highly porous materials with high connectivity, such as mushrooms, are insufficiently developed (van der Sman et al., 2024). Therefore, an empirical approach was used with the assumption of ideal shrinkage of the solid (hyphae) phase and an empirical relationship between the porosity and moisture content (dry basis). The drying model was fitted to the experimental drying data at temperatures of 33 °C, 38 °C, and 51 °C. The model was validated by experiments performed at 35 °C. Furthermore, to validate the thermodynamic states of the two distinct water phases, which are critical for understanding cell membrane integrity, T_2_-NMR measurements were performed on mildly dried mushroom samples. This experimental insight allowed the drying model to be applied to interpret earlier T_2_ relaxation data for mushrooms dried at 30 °C, 40 °C, and 50 °C (Qiu et al., 2021). The T_2_-NMR data, which revealed the presence of one or two water populations, were integrated into an earlier state diagram based on conductivity measurements. This combined analysis helped identify the critical conditions associated with the loss of cell membrane integrity.

## 2 Theory model for aw prediction of viscoelastic mushroom

In this section, an improved theoretical model that accounts for viscoelastic properties was introduced to predict a_w_. This model also describes the viscoelastic relaxation modes of mushrooms during drying.

### 2.1 The thermodynamic phase-separated system of mushroom

**Fig. 1:**
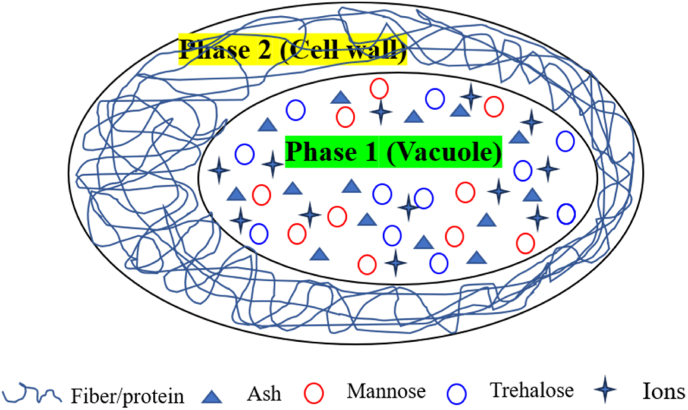
The thermodynamic phase-separated system of hypha in mushroom.

Mushrooms consist of hyphae, which resembles plant cells, containing cell walls and vacuoles. Water is partitioned among the cell wall, cytoplasm, and vacuole. As shown in Fig. 1, the hypha of a mushroom is approximated as a thermodynamic phase-separated system, following (Jin et al., 2014). All solutes, including sugars (such as mannitol and trehalose) and ions, were localized in the vacuole and designated as **Phase1**. In contrast, all biopolymers, such as proteins in the cytoplasm and fibers in the cell wall, were assumed to reside in **Phase 2**. In principle, the cell wall and cytoplasm are separated by the cell membrane. However, as the cytoplasm and cell wall consist of biopolymer networks, they can be aggregated into a single phase with the same water activity ($a_{w}$). These two phases are separated by the cell membrane (of the vacuole), which allows water to diffuse between but prevents solute transport, provided that the membrane remains intact. Likewise, a local equilibrium can be achieved between the vacuole and biopolymer phases, with water partitioned over the two thermodynamic phases, such that their water activities are equal. Therefore, the water activity of mushrooms with intact cell membranes is as follows:

(1) $$a_{w,1}=a_{w,2}$$

The water activity in each phase was calculated based on the composition of the respective phases. The main compounds in *shiitake* mushrooms have been reported in the previous work (Hu et al., 2023), and their details are given in Table S1 in the **Supplementary Materials (**Section 1.1**)**. The fraction of water in each phase was determined via minimization procedures solved for the partitioning of a fixed amount of water, which adhered to the condition a_w,1_ = a_w,2_.

The chemical potential of water in each phase, ${\Delta \mu }_{w,i}$, can be expressed as:

(2) $${\Delta \mu }_{w,i}=RTlna_{w,i}={\Delta \mu }_{w,mix,i}+v_{w}p$$

where i indicates Phase1 (vacuole) or Phase 2 (cytoplasm and cell wall). The term ${\Delta \mu }_{w,mix,i}$ varies in each phase owing to differences in composition, following the Flory-Huggins theory. The constitutive relations and parameters for ${\Delta \mu }_{w,mix}$ have been provided in the previous publication (Hu et al., 2023). In contrast to existing moisture sorption models, an extra term, $v_{w}p$, is incorporated to account for the influence of osmotic pressure ($p,J/m^{3}$). This pressure arises from the attraction of water by the solutes present in the vacuole. $v_{w}$ (m^3^/mol) is the molar volume of water. The consequent cell volume expansion is hindered by the elastic cell wall, which encapsulates the cells, including the outer cell membrane. The cell wall is stretched, which renders mechanical stress and counter (turgor) pressure acting against cell expansion. At equilibrium, the turgor pressure is equale to the osmotic pressure inside the cell. Hence, in the case of phase2, $v_{w}p=-{\Delta \mu }_{elastic}$ is assumed owing to the elastic stress. Assuming isotropic deformation, the stress is represented by a scalar $\Pi_{elastic}$, yielding the stress tensor $\sigma =-\Pi_{elastic}\;I$ (I = identity tensor). The elastic chemical potential change is ${\Delta \mu }_{elastic}=\nu_{w}\Pi_{elastic}$ ($\nu_{w}$ = molar volume of water). Based on the Flory-Rehner theory, $\Pi_{elastic}$ depends on the relative volume change $J=\frac{1}{\tilde{\varphi }}=\frac{\varphi_{s}}{\varphi_{ref}}$ ($\varphi_{s}$ = polymer volume fraction, $\varphi_{ref}$ = reference state) (van der Sman, 2023). For non-uniform large deformations, the displacement is characterized by the stretch parameter $\lambda_{i}$ (total deformation in principal direction i). Simplifying with isotropic cell wall deformation and hydrogel incompressibility, $\frac{1}{\tilde{\varphi }}=\lambda^{3}$ ($=\lambda_{i}$). Note, at reference state (λ=1; $\varphi_{s}=\varphi_{ref}$), the viscoelastic stress σ vanishes (van der Sman, 2023).

### 2.2 Viscoelastic relaxation modes for mushrooms

The elastic modulus and viscoelastic behavior of mushrooms depend on both moisture content and temperature (Hu et al., 2022). Yet *shiitake* tissue at 15 % moisture (wet basis) shows a glass-transition temperature below −40 °C (Zhang et al., 2024; Zhao et al., 2016), which is far below the drying and sorption temperatures considered here. Therefore, temperature-induced changes in the modulus are neglected in this study. The elastic stress is assumed to relax over a spectrum of times. Although fractional-derivative and Marin–Graessley formulations can describe the stress (van der Sman, 2023; Xiao et al., 2016), a simplified dual-mode Maxwell model was adopted, supported by compression tests on mushroom tissue dried at 35 °C (Hu et al., 2023).

Because the experiments were constrained by moisture content, and there was insufficient data in the dry regime, the regression functions for the viscoelastic properties were extrapolated to a wider regime. Furthermore, viscoelastic properties are measured at the scale of the whole mushroom, but the viscoelastic properties relevant for sorption are those of the cell wall, which are probably higher due to the presence of fibers. Nevertheless, the dependencies on moisture and temperature are assumed to be comparable to those of the viscoelastic properties at the tissue scale. Two scaling factors, F_Gi_, and F_τi_, used for fitting to the dynamic vapor sorption measurements were introduced. Note, these fittings were performed independently of the fitting of the drying model to the drying experiments.

Following (van der Sman, 2023), the Maxwell model was extended to finite strains. The implementation details are given in the **Supplementary Materials (**Section 1.2**).** The Maxwell model has two relaxation modes, slow and fast. The relaxation of each mode is governed by :

(3) $$\frac{{d\phi }_{ref,i}}{dt}=\frac{\phi_{s}-\phi_{ref,i}}{\tau_{0,i}}$$

where $\phi_{ref,i}$ is the internal variable for each relaxation mode, and $\tau_{0,i}$ is the relaxation time, which is moisture content- and temperature-dependent.

The total stress σ is the sum of the contributions from both the relaxation modes:

(4) $$\sigma =\sum_{i}G_{i}*\left({\tilde{\varphi_{i}}}^{\frac{1}{3}}-\tilde{\varphi_{i}}\right)$$

where $G_{i}$ is the elastic modulus for each mode, and $\tilde{\varphi_{i}}=\frac{\phi_{s}}{\phi_{ref,i}}$ represents the relative volume change for each mode (i = 1,2 for slow and fast modes, respectively).

## 3 Physical and mathematical model for drying process

### 3.1 Model assumptions

**Fig. 2:**
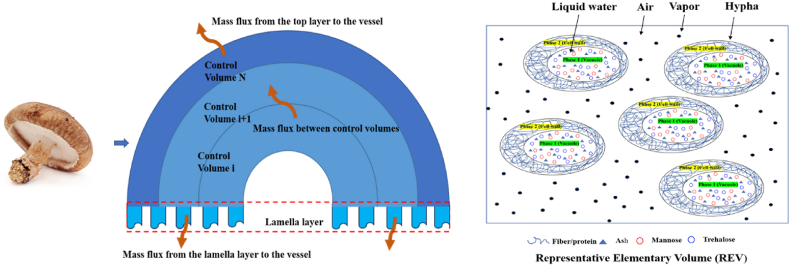
Schematic of the division of the mushroom geometry in control volumes as used in the drying model and representation of multiple phases in the REV.
5) The heat and mass transfer during drying are primarily driven by convective airflow. Due to the mild drying temperature and long drying time, the mass transfer mechanism for water in mushrooms is assumed to include a) diffusion of liquid water within the matrix, following Fick's law, and b) the vapor transport by diffusion in the gas part (pores) of mushroom (Castro et al., 2018; Defraeye and Radu, 2018).
6) The moisture diffusion coefficient $D_{w_{diff}}$ is based on the theory of (van der Sman and Meinders, 2013), as detailed in Supplementary Materials.

Based on the established framework of multiphase porous media transport phenomena, the drying model of *shiitake* mushroom (with a weight of 25 × 10^−3^ kg) was developed under the following assumptions:

1) Mushrooms are treated as porous media, consisting of hyphae (including liquid water and solid) and pores filled with air and vapor.
2) Both the hyphae and pore spaces are continuous phases.
3) Air and vapor are ideal gases.
4) The geometry of mushrooms is simplified as a hemisphere, and the consequent drying experiments are thus performed with mushrooms with stipe removed. As shown in Fig. 2, the mushroom is geometrically discretized into two distinct components: a hemispherical cap and a lamella layer. The cap is composed of a hyphal matrix interspersed with air-filled pores, and the lamella layer is modeled as a dual-porous structure characterized by air gaps between the porous lamellar tissues. The air gaps within the lamella account for 50 % of the total volume of this dual-porous region (i.e., $\varphi_{hypha,lam}=\varphi_{air,lam}=0.5$). The intrinsic material properties of the lamellar matrix are assumed to be consistent with those of the cap. These air gaps are excluded from the mass and energy balance calculations and are assumed to be explored under the same environmental conditions as the external drying atmosphere.

### 3.2 Governing equations

#### 3.2.1 Mass equation

The total moisture concentration $c_{moisture}$ is defined as:

(5) $$c_{moisture}=\left({1-\varphi }_{air}\right)c_{w}+\varphi_{air}c_{v}$$

where $\varphi_{air}$ is the porosity. $c_{w}$ (kg/m^3^) is the moisture concentration of the hyphae. $c_{v}$ (kg/m^3^) is the vapor concentration in the pores, which is assumed to reach the equilibrium state, and hence can be calculated with $c_{v}={a_{w}M}_{v}\frac{p_{v,sat}\left(T_{p}\right)}{RT}$. The mass change of the total moisture is given by:

(6) $$\frac{\partial c_{moisture}}{\partial t}=-\nabla \cdot \left(j_{w,diff}+j_{v,diff}\right)$$

where $j_{w,diff}$ and $j_{v,diff}$ (kg·s^−1^·m^−2^) are the liquid water and vapor mass flux density, respectively.

(7) $$j_{w,diff}=-F_{w,diff}\left({1-\varphi }_{air}\right)D_{w_{diff}}\nabla c_{w}$$

(8) $$j_{v,diff}=-{\varphi_{air}D}_{{gas}_{vap}}\nabla c_{v}$$

$D_{w_{binary}}$ and $D_{{gas}_{vap}}$ are the diffusion coefficients of water and vapor, respectively.

$D_{w_{diff}}$ follows the theory of (van der Sman and Meinders, 2013), which has been shown to be valid for carbohydrate solutions. If this theory holds, cell wall material (CWM) and mannitol can be regarded as carbohydrates, as they are the main dry matter in mushroom. In the dry state, the predictions are largely independent of the molar weight of the carbohydrate, but in the wet regime, they depend on the molar weight. To account for the possible mismatch between the theoretical and actual moisture diffusion in the wet regime, the theoretical diffusion coefficient is multiplied by a constant factor $F_{w,diff}$, to be estimated during model fitting. $F_{w,diff}$ is assumed to be of the order of 1, and hence 0.1< $F_{w,diff}$ <10. The detailed expression for $D_{w_{diff}}$ is given in the **Supplementary Material (**Section 1.3**)**.

#### 3.2.2 Energy equation

The energy balance accounts for the heat convection due to water vapor and heat conduction through the mushroom.

(9) $$\frac{\partial q}{\partial t}=-\nabla \cdot \left(j_{w}c_{p,w}\left(T-T_{0}\right)+j_{v}c_{p,v}\left(T-T_{0}\right)+j_{v}h_{evap}\right)+\nabla \cdot \left(\lambda_{eff}\nabla T\right)$$

(10) $$q=\left(\varphi_{s}\rho_{s}c_{p,s}+\varphi_{w}\rho_{w}c_{p,w}{+\varphi }_{air}c_{v}c_{p,v}\right)T{+\varphi }_{air}c_{v}h_{evap}$$

where q (J/m^3^) is the energy density, T (K) is the product temperature, and Zero Celsius $T_{0}$ (K) is taken as the reference temperature for the enthalpy; $j_{i}$ (kg/(m^2^·s)) indicates the mass flux for liquid water and vapor, and $c_{p,w}$, $c_{p,v}$ (J/kg/K) indicates the specific heat capacity of water and vapor, respectively. $h_{evap}$ (J/kg) is the enthalpy of vaporization.

### 3.3 Boundary conditions

The boundary conditions for moisture and heat transport are as follows:

(11) $$j_{w,diff}+j_{v,diff}={F_{w,conv}h}_{ext}\left(c_{v,surf}-c_{air}\right)$$

(12) $$\lambda_{mushroom,eff}\nabla T=h_{ext}\left(T_{air}-T\;\right)-{△H}_{evap}\beta_{ext}\left(c_{v,surf}-c_{air}\right)$$

In Eq. (11), $c_{v,surf}=a_{w}\;c_{sat}\left(T_{surf}\right)$ is the vapor concentration at the boundary layer, which is in equilibrium with the tissue at surface having a water activity of *a*_*w*_. $c_{sat}$ is the vapor concentration at saturated vapor pressure, $T_{surf}$ indicates the surface temperature of mushroom. $c_{air}$ is the vapor concentration in the air. In Eq. (12), the term $h_{ext}\left(T_{air}-T\right)$ refers to the heat transfer from the air to mushroom on the boundary layer following Fourier's law. The term ${△H}_{evap}\beta_{ext}\left(c_{v,surf}-c_{air}\right)$ accounts for the evaporative cooling, where ${△H}_{evap}$ is the enthalpy of evaporation. The external heat transfer coefficient $h_{ext}$ (W·m^−2^·K^−1^) is the sum of the coefficients of heat radiation ($h_{rad}$) and heat convection ($h_{conv}$). $h_{rad}$ follows from the linearization of the Stefan's law, and $h_{conv}$ is expressed with a convenient rule of thumb for heat transport in air at (nearly) flat surfaces (ASHRAE, 1997):

(13) $$h_{rad}=e*4*\sigma *T_{wall}^{3}$$

(14) $$h_{conv}=6+10*u_{air}$$

The wall of the drying oven is assumed to have an emissivity of e which is unknown and needs to be estimated. For a perfect black body e=1, the range of e is estimated to be between 0.1 and 0.9. σ is the Stefan constant with the value of 5.70 × 10 ^−8^ (W·m^−2^·K^−4^). $T_{wall}$ is equal to $T_{air}$, as the absolute temperature in Kelvin of the walls in the drying oven. $u_{air}$ (m/s) refers to the velocity at the surface of mushroom, which was estimated in this study. Normally, the external mass transfer coefficient $\beta_{ext}$ is assumed to be largely related to the convective heat transfer coefficient $h_{conv}$ via the Lewis relationship: $\beta_{ext}=F_{w,conv}\frac{h_{conv}}{\rho_{air}c_{p,air}}$. $F_{w,conv}$ accounts for possible deviations caused by: 1) temperature gradients across the boundary layer that can give rise to deviations in the Lewis relation. 2) due to the porosity, the effective surface area might also be slightly larger. 3) internal porosity can generate small convective/pressure driven flows (also enhancing the diffusion), and these convective flows leaving the mushroom surface can thin the boundary layer.

In the **Supplementary Materials (**Section 1.3-1.4**),** the material properties and model parameters are provided, as well as the details of the numerical implementation of the mathematical model.

## 4 Material and methods

In this paper, material preparation, measurement of moisture content and product temperature of mushroom as well as the air temperature and relative humidity monitoring during drying, measurement of dynamic moisture sorption of mushroom are given in the **Supplementary Materials (**Section 2**).**

### 4.1 NMR measurement and analysis

A mushroom cap was cut into four equal parts with a blade in a crisscross pattern, and used one of them to conduct the NMR measurement and analysis. A custom-built Niumag Pulsed NMR analyzer (Suzhou Niumag Analytical Instrument Corporation, Suzhou, China) was used, featuring a 0.5 T permanent magnet that corresponds to a proton resonance frequency of 23.2 MHz at 32.00 ± 0.02 °C, with 90° and 180° pulses set at 16 ms and 33 ms, respectively. A hot air drying device was positioned beneath the magnet, enabling upward air flow to facilitate the online NMR measurements during the drying process. The quartered sample was placed at the center of an NMR tube (outer diameter: 30 mm) and dried at a set temperature of 35 °C for 10 h, during which the online NMR measurements were conducted.

The transverse relaxation times (T_2_) were determined using Carr-Purcell-Meiboom-Gill (CPMG) pulse sequences. The main parameters for T_2_ relaxation time measurements were TW (time waiting) = 2000 ms, TE (time echo) = 0.5 ms, NECH (number of echoes) = 12,000, NS (number of scan) = 4. Logarithmic coordinates of raw data were used to construct the T_2_ distribution curves with multi-exponential model under the program of the MultiExp Inv Analysis (Suzhou Niumag Analytical Instrument Corporation, Suzhou, China).

### 4.2 Relative humidity reconstruction for model parameters fitting

During drying modeling, parameters estimation was performed by fitting simulated moisture contents and center temperatures of HA33, HA38, and HA51 against experimental data obtained from the previous study (Hu et al., 2022). Due to the constraints of the experimental conditions at that time, it was impossible to simultaneously measure the variations in relative humidity during these drying processes. In this study, drying experiments at T = 35 °C (HA35) was performed, where the relative humidity was monitored simultaneously. This dataset was then used to validate the fitted model. The drying oven used for HA33, HA38, and HA51 is the same as for HA35 in this study. The absolute humidity in different drying experiments was assumed to be comparable and the relative humidity data can be reconstructed accordingly. This hypothesis was tested with supplementary experiments of HA33, HA38, and HA51, where relative humidity was monitored using the same method as for the HA35 experiments.

### 4.3 Parameter fitting and statistical analysis

For moisture sorption model, the unknown factors are $F_{G0}$, $F_{G1}$, *F* $\tau_{0_{0}}$, and ${F\tau }_{0_{1}}$, as described in viscoelastic property of mushroom in **Supplementary Materials (**Section 1.3**)**. This fitting was accomplished manually by comparing the experimental data (the desorption branch during the first sorption-desorption cycle and the adsorption branch measured during the second cycle) and the calculated results.

The drying model is based on existing predictive theories as much as possible. The remaining unknown factors are as follows: the emissivity e , $F_{w,diff}$ and $F_{w,conv}$, which are estimated by fitting simulated moisture contents and center temperatures to those of hot air drying experiments performed at T = 33, 38, and 51 °C (HA33, HA38, and HA51) obtained from our previous study (Hu et al., 2022).

Fitting was performed by minimizing the objective function with the squared difference between the predicted and simulated temperatures and moisture content. The objective function is expressed as follows:

(15) $$LSE\_sum=\sum_{i}\left(\left(\frac{\sum_{j}{\left(\Theta_{p,\exp}-\Theta_{p,pred}\right)}^{2}}{N_{T_{p,\exp}}}\right)+10\left(\frac{\sum_{j}{\left(y_{w,\exp}-y_{w,pred}\right)}^{2}}{N_{y_{w,\exp}}}\right)\right)$$

where i indicates the experiment (HA33, HA38, and HA51), and j indicates their datapoints. $T_{p,\exp}$ and $T_{p,pred}$ represent the experimental and simulated temperatures respectively, while $y_{w,\exp}$ and $y_{w,pred}$ denote the experimental and simulated moisture contents, respectively. As both the temperature and moisture content contribute to the sum of squares, the values must be properly rescaled to variances between 0 and 1. The mass fraction is already properly scaled, but the temperature is reduced using the air temperature and the initial product temperature: Θ = (T_p_ – T_init_)/(T_air_-T_init_). As the number of data points for the temperature and moisture content recordings differed significantly, the sum of squares was rescaled by their respective numbers of data points: $N_{Tp,\exp}$ and $N_{yw,\exp}$. Because of the particular importance of moisture content on the prediction, a weight factor of 10 for the least squares error of moisture content was applied. In the initial stages of fitting, this weight factor was modified to make the objective function sensitive to its temperature and moisture variations similar. Because of the strong nonlinearity of the problem, the fitting process was performed manually.

To identify the optimal model parameters values that minimize the response variable (LSE_sum), a sequential single-factor optimization approach was employed. In each simulation series, one parameter (either e, $F_{w,conv}$​, or $F_{w,diff}$) was varied while keeping the other parameters constant. The initial parameter ranges were chosen based on preliminary studies and relevant physical constraints. The corresponding LSE_sum values were recorded for each simulation. After each series, the parameter combination yielding the lowest LSE_sum was selected as the new working point for the next round of single-factor variations. This iterative refinement process was repeated until convergence was achieved—i.e., until further iterations did not yield a lower LSE_sum, indicating a local minimum of the objective function. A detailed description of the parameter refinement procedure is provided in the **Supplementary Materials (**Section 1.5**)**.

The parameter fitting goodness and validation accuracy for the drying model was evaluated using statistical indicators, including the coefficient of determination (R^2^), root mean square error (RMSE), and mean absolute error (MAE). The equations and meanings of the statistical indicators are provided in the **Supplementary Materials (**Section 1.6**)**.

## 5 Results and discussion

### 5.1 NMR results

**Fig. 3:**
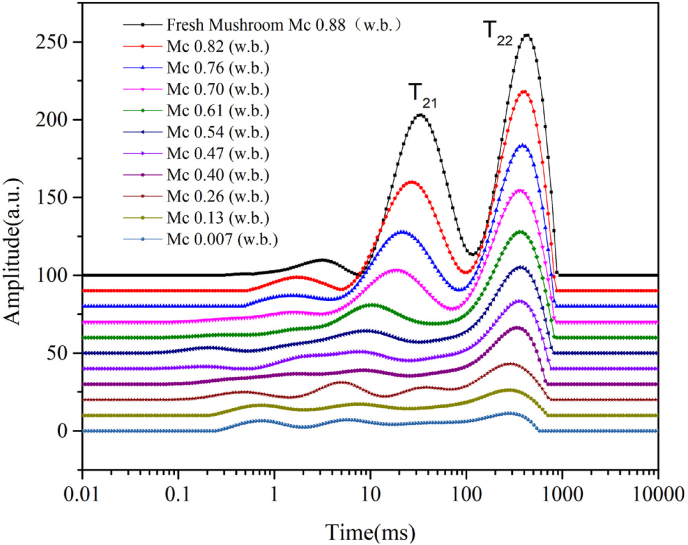
The transverse relaxation curves (T_2_) of fresh shiitake mushrooms during the HA35 drying within 10 h.

The goal of the NMR measurement during drying at 35 °C on fresh mushrooms is to examine the viability of the hypothesis-put up in a prior work by (Hu et al., 2023) - that the mushroom has two distinct thermodynamic phases. As demonstrated in Fig. 3, two main peaks, denoted as T_21_ and T_22_, are observed during the entire drying process. The T_21_ and T_22_ peaks have been used to identify water fractions in different cell compartments of cellular food materials such as broccoli and mushroom (Li et al., 2021; Qiu et al., 2021; Xu et al., 2017). The T_2_ peaks are attributed to water populations with different molecular environments. The T_21_ peak, exhibiting T_2_-relaxation times ranging from 10 to 100 ms, signifies water immobilized within the cell wall and cytoplasm. This immobilization is attributed to the bonding of water with biopolymers, such as proteins and polysaccharides, which restricts its mobility. The T_22_ peak, whose relaxation time spans from 100 to 1000 ms, signifies the presence of highly mobile water within the vacuole. This water is typically accompanied by small molecules such as sugars or minerals (Li et al., 2021; Qiu et al., 2021; Xu et al., 2017). Therefore, the NMR results presented in Fig. 3 confirm the presence of two distinct thermodynamic phases in mushrooms throughout the drying process.

Furthermore, it was observed that the amplitudes of both T_2_ peaks decreased during drying, and shifted to lower relaxation times. This indicates a reduction in the mobility of water within both phases towards the end of drying, which is a consequence of the decreasing moisture content relative to the fixed amount of solids, as well as the increase in concentrations of solutes in vacuoles. Toward the end of drying, there were no distinct populations, which could be due to the redistribution of water within the two main phases during hot air drying. When the cell membrane loses its integrity, free water in the vacuole is transformed into immobile water through bonding to carbohydrates (Li et al., 2021). It can be deduced that these two main water populations eventually mixed, and exhibited a single T_2_ peak (Cheng et al., 2020).

### 5.2 Moisture sorption prediction of mushroom with improved flory huggins theory model

**Fig. 4:**
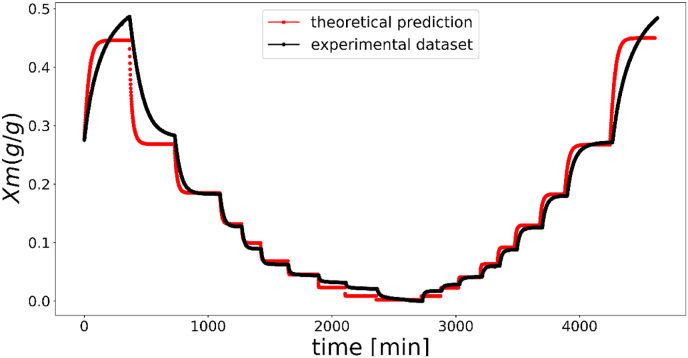
Comparison between simulation and experiment of dynamic moisture sorption of HA35 dried mushroom powder (The left and right branches indicate desorption and absorption processes during the first and second sorption–desorption cycles, respectively).

Parameter fitting was performed for the dynamic vapor sorption response of a mushroom sample, and the results are presented in Fig. 4. The parameter values that best fit the experimental data were: $F_{G0}=0.1$; $F_{G1}=3\;;\;{F\tau }_{0_{0}}=10$ ; *F* $\tau_{0_{1}}=100$.

Overall, the simulated moisture content values at the end of each relative humidity (RH) step are largely in agreement with the experimental data collected in the previous work (Hu et al., 2023), and the simulated curves present good fitting with the experimental ones, particularly in the range 0.2 < RH < 0.8. However, for the desorption branch of first cycle (left branch in Fig. 4, data shown in previous paper), it is noted that the steady state experimental values are not attained at the end of the ‘equilibration time’, when the relative humidity (RH) is between 0.1 and 0.2 or higher than 0.8. The experimental moisture content values were higher than those predicted by simulation. This difference can be attributed to the failure of dried samples to reach the true equilibration at RH = 0.1–0.2, due to the stresses locked within the matrix after drying (van der Sman, 2023). The viscoelastic relaxation time of dry mushrooms is much longer than the practical time scales during DVS measurement, and the mechanical stresses cannot be effectively relaxed, which impedes the attainment of a true equilibrium. Additionally, slow diffusion is another significant contributing factor. On the other hand, during moisture sorption at RH > 0.8, the intact cell membranes of HA35 dried samples can restore the water holding capacity in the vacuole and thus increase the turgor pressure via stretching of the cell walls. Consequently, this physical process necessitates a longer period to achieve equilibration, as reported in the previous work (Hu et al., 2023).

**Fig. 5:**
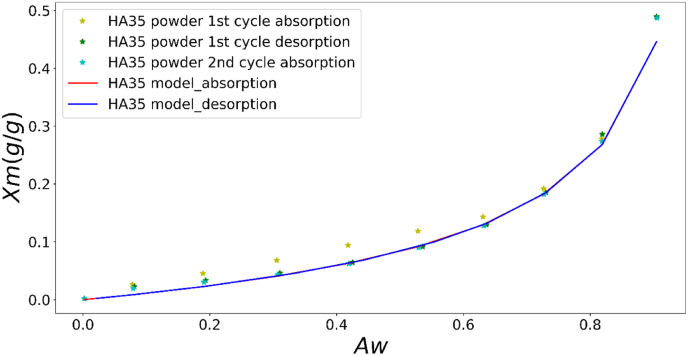
Comparison between simulation and experimental MSI of HA33 dried mushroom.

When modeling the drying process, it is suspected that stress relaxation affects water sorption. In Fig. 5, it shows the simulation results of the moisture sorption isotherms with the comparation with experimental data obtained in the previous study (Hu et al., 2023). The experimental moisture sorption isotherm (MSI) measured during the first cycle exhibits a distinct hysteresis phenomenon, which is barely noticeable from the second cycle onwards. This observed hysteresis can be attributed to the stress accumulated in the matrix during the rapid drying process of the samples. When the dried mushroom samples are exposed to a prolonged period of water absorption during the first cycle of MSI measurement, the internal stress within the samples gradually dissipates over this ample time span. As a result, the hysteresis disappears when the samples are subjected to the second cycle of MSI measurement. However, this hysteresis phenomenon is not addressed in this analysis, as the drying history of the freeze-dried sample is unknown. Its drying rate was definitely faster than during DVS experiments, leading to the locking-in of stresses. Furthermore, the information on the elastic modulus in the dry regime is lacking, which could not be obtained with the compression test (Hu et al., 2023).

Nevertheless, these results demonstrate that the satisfactory fitness allows us to utilize the updated moisture sorption theory model that accounts for viscoelastic relaxation to develop a drying model for mushrooms.

### 5.3 The analysis of porosity evolution of mushrooms during drying

Moisture loss during the drying process leads to shrinkage of the mushroom, causing a change in its porosity. In principle, accurately determining these changes in porosity necessitates the resolution of the momentum balance. However, such computations are complex and demanding. Instead, a simulation of the porosity evolution of mushrooms was presented by utilizing an empirical relation derived from the shrinkage ratio curves measured during the drying process. The detailed simulation process for predicting the porosity evolution based on the empirical relation is given below.

As depicted in Fig. 2, the mushroom exhibits a porous structure comprising hyphae (consisting of solid material and liquid water) and gas within the matrix (containing vapor and air). The solid, liquid water and gas fractions of the hyphae are distinguished. Due to the fact that the amount of solid material remains constant during drying and the amount of liquid water is governed by the mass balance, the gas fraction (porosity) is determined by the removal of water (in vapor form) and air from the matrix.

Hence, the volume fractions undergo changes as water and air are lost. The variation in the gas phase (porosity) was represented as the change in the volume ratio between the air and solid material within the mushroom, and the derivation is given in the **Supplementary Material (**Section 3.1**)**:

(16) $$\frac{V_{mushroom}\left(t\right)}{V_{solid}}=\frac{V_{air}\left(t\right)}{V_{solid}}+1+\text{Xm}\left(t\right)*\frac{\rho_{solid}}{\rho_{water}}$$

The mushroom volume change $V_{mushroom}\left(t\right)$, can be derived from an empirical relationship that describes the change in shrinkage ratio during hot air drying. Subsequently, the porosity evolution can be expressed by the relationship between the air volume fraction (relative to the constant solid volume) and moisture content (d.b.): $\frac{V_{air}}{V_{solid}}$ ∼ Xm

**Fig. 6:**
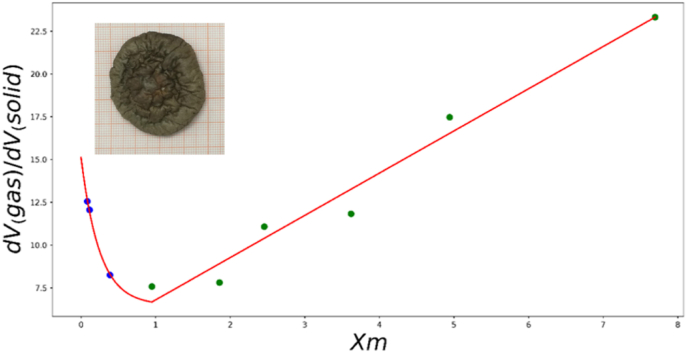
Volume fraction ratio of gas and solid phases change of mushroom during HA35.

To establish the relationship between $\frac{V_{air}}{V_{solid}}$ and $X_{m}$, exponential and linear fitting methods was applied to analyze the experimental data, as shown in Fig. 6.

When $X_{m}<0.95$,

(17) $$\frac{dV_{gas}}{dV_{solid}}=8.637e^{-4.034*\text{Xm}}+6.674$$

when $X_{m}>0.95$,

(18) $$\frac{dV_{gas}}{dV_{solid}}=2.467*X_{m}+4.33$$

Subsequently, this empirical relation was incorporated into the modeling of hot air drying to account for the impact of mushroom shrinkage on heat and mass transfer.

From the porosity (air volume fraction) curve shown in Fig. 6, it is observed that the porosity increases suddenly at the end of drying. This phenomenon is also observed by (Madiouli et al., 2007; Nguyen et al., 2018). According to (Gulati and Datta, 2015; Nguyen et al., 2018; van der Sman et al., 2024), it can be attributed to the formation of a tough skin, meanwhile the inside is soft, undergoing a mechanical (cavitation) instability due to the shrinkage of the core. At the late stage of the drying process, as depicted in the mushroom image in Fig. 6, the surface layer stiffens upon drying. The wrinkled appearance on the surface serves as evidence of the mushrooms' case-hardening. Simultaneously, as drying progresses, the rubbery matrix in the core region shrink, leading to a sudden rise in porosity.

### 5.4 Model fitting and validation

Following the fitting of the moisture sorption model, the drying model was fitted to experiments via minimizing the objective function (Eq. (15)). The detailed fitting procedure and results of all parameter combinations are given in Table S2 & Table S3 in the **Supplementary Material (**Section 1.5**),** showing that the best fit is obtained for e = 0.9, $F_{w,conv}$ = 5 and $F_{w,diff}$ = 8. All correction factors are in the expected range 0.1<F_i_ < 10

**Fig. 7:**
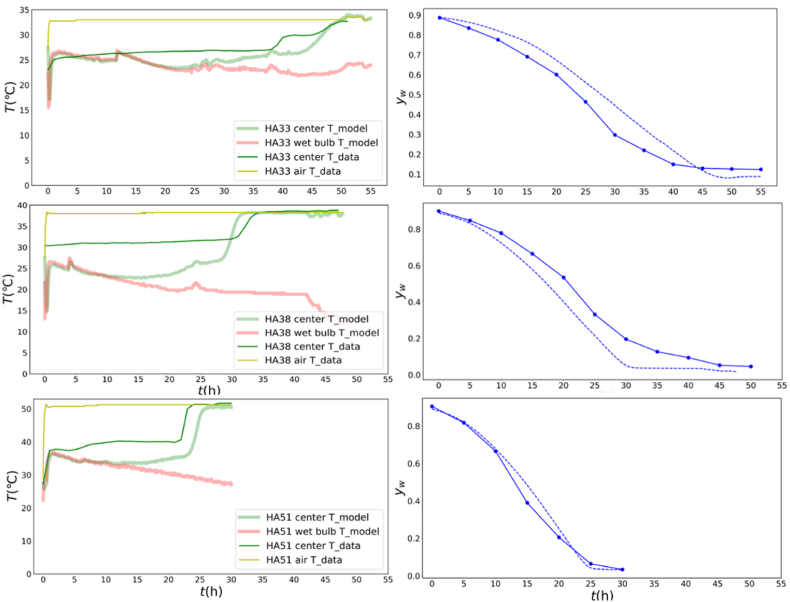
Experimental and simulated temperatures and moisture contents of mushrooms dried by HA33, HA38, HA51 with the optimal parameter combination.

Fig. 7 presents the final fitting results for HA33, HA38, and HA51 with the optimal parameter combination. The moisture content curves fit well with the simulated ones. The coefficients of determination (R^2^) for HA33, HA38, and HA51 are 0.9361, 0.9325, and 0.9849, respectively. The mean absolute errors (MAEs) are 0.068, 0.078, and 0.030, respectively. The root mean square errors (RMSEs) are 0.08205, 0.09371, and 0.04576, respectively. All experimental temperature curves ran below their simulated counterparts until the final drying stage. Yet, the moment of the rapid temperature rise is captured quite accurately for all experiments, which is probably driven more by the kinetics of moisture transport. This systematic deviation might arise from the fact that the relative humidity at these three temperatures were not directly measured, even though the environmental absolute humidity in the oven remained almost constant across different experimental periods, as shown in Fig. S3 in the **Supplementary Materials (**Section 3.2**)**. The actual relative humidity for HA33, HA38, and HA51 might have been lower than that of the auxiliary measurements taken later, which could be the main source of error. A consistent pattern is also observed in which all center temperatures stabilized near the wet-bulb temperature for a long time (approximately 25 h for HA33,10 h for HA38,12 h for HA51) and rapidly increase toward the air temperature at the late drying stage. This trend is primarily driven by internal evaporative cooling within the porous mushroom structure. If sufficient moisture is present, this cooling effect persists and maintains the central temperature near the wet-bulb level. However, when drying nears completion, with little moisture remaining in the core, the evaporation front shifts to the mushroom center, and the cooling effect diminishes, allowing the temperature to rise quickly toward the air temperature.

**Fig. 8:**
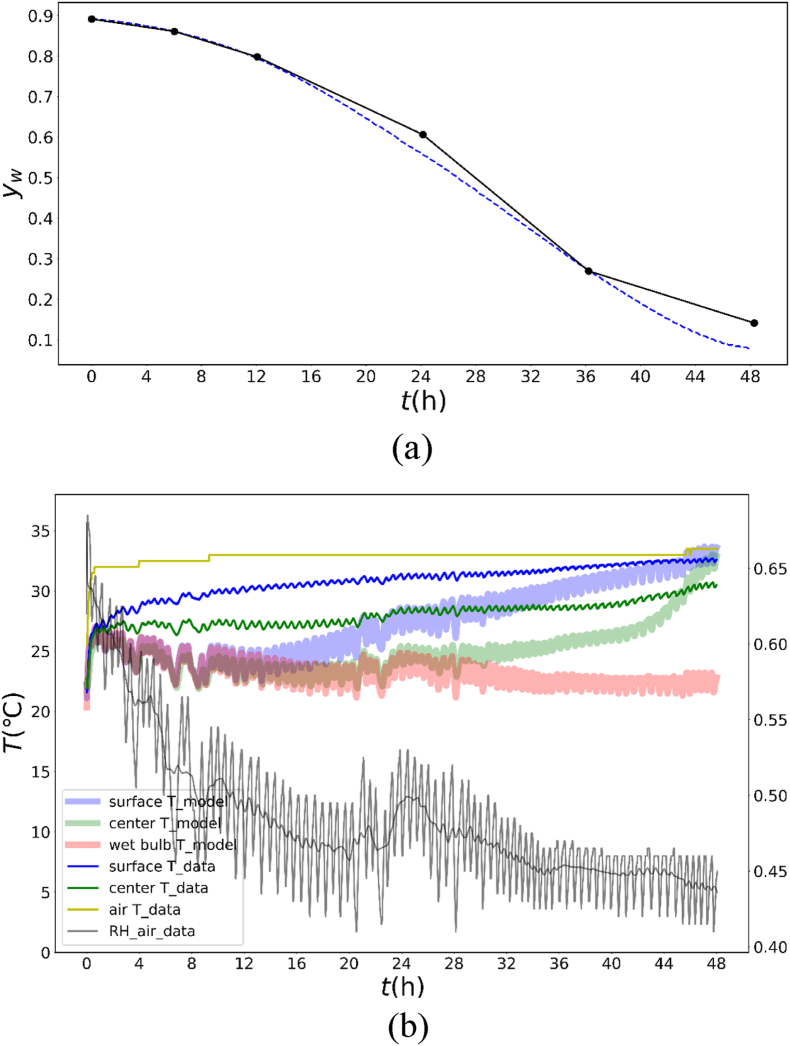
Experimental and simulated moisture contents (a) and surface and center temperature evolution (b) of mushrooms dried by HA35 with the optimal parameter combination.

For model validation, a simulation of moisture content and surface and center temperatures of the mushroom during hot air drying at 35 °C (HA35) was conducted with the established model, and the results were compared with the experimental data in Fig. 8. As shown in Fig. 8a, the simulated average moisture content across the entire sample layer exhibited strong agreement with the HA35 experiment, as validated by high coefficients of determination (R^2^ > 0.9875), low mean absolute errors (MAEs = 0.02205), and low root mean square errors (RMSEs = 0.03656), confirming the reliability of the model for moisture content prediction.

As shown in Fig. 8b, the model reasonably predicts the overall trends and final values of the surface and center temperatures; the simulated temperatures still consistently exhibit a lower bias than the experimental data, similar to Fig. 7, although the air temperature and relative humidity were monitored synchronously with the product temperatures. Possible causes include the following:

a) Due to thermal contact limitations, thermocouple probes may not have perfect contact with the mushroom, failing to capture the exact local conditions, especially in the internal region. When measuring the surface temperature, the probes are additionally influenced by convective heat transfer from the surrounding hot air, which introduces measurement errors. Specifically, as shown in Fig. 7 and Fig. 8b, the simulated temperatures are consistently lower than the experimental temperatures. This discrepancy might arise because the model outputs a simulated value at a single specific point, whereas the temperature sensor measures the temperatures across multiple points on the surface and outputs an average value. Mushroom shrinkage and case-hardening during drying result in geometric inconsistencies between the experimental sample and simulation model (Zeineldin, 2008).
b) Simplified heat/mass transfer boundary conditions

The model assumes idealized surface conditions and overlooks the impact of non-uniform airflow on convective heat transfer coefficients. These airflow irregularities arise from the mesh structure supporting the mushrooms, which either conducts or blocks the airflow, thereby introducing variability in heat transfer that the model does not account for. Real-world air temperature/distribution variations around the mushroom create localized temperature differences that are unaccounted for in simulations, contributing to higher experimental readings.

Furthermore, it is observed that the surface and internal temperatures fluctuate upward as heat transfers inward in Fig. 8. Surface temperature fluctuations mirrored thermodynamic equilibrium adjustments between aw and RH, while center temperature fluctuations arose from evaporative cooling in the porous structure, modulated by RH-dependent evaporation rates. RH fluctuations originate from dryer on/off control, consistent with observations in apple disks intermittent microwave drying by (Kumar et al., 2016). It is also observed that the surface and center temperatures stabilized at quasi-steady wet-bulb temperature for ∼12 and 30 h respectively, which might be directly correlated with its sustained high aw, as ongoing internal evaporation maintained the cooling effects. To determine the physical mechanism of mushroom drying, the moisture and temperature distributions was simulated and analyzed, as described in the next section.

### 5.5 Drying mechanism analysis

#### 5.5.1 Water activity distribution

**Fig. 9:**
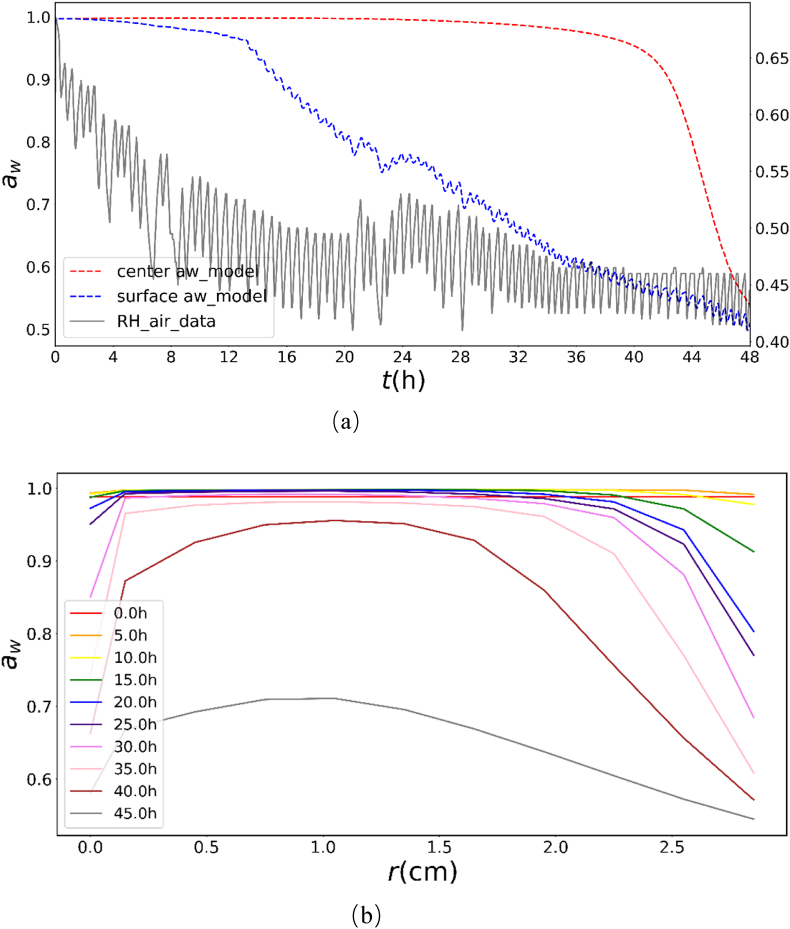
Surface and center aw change during HA35 (a) and aw distribution evolution at different drying time intervals (b).

Water activity (aw) evolution provides insights into the evolution and distribution of moisture content and links the relationship between moisture and temperature dynamics. In Fig. 9a, the surface aw decreases as drying progresses, in accordance with the reduction in the relative humidity (RH). In contrast, the central a_w_ remains approximately unity until the final drying stage, a pattern directly tied to the prolonged maintenance of low central temperatures owing to internal evaporative cooling. Fig. 9b presents the non-uniform spatial distribution of a_w_ at different time intervals during the drying process: layers near the cap surface (r = 3.0 cm) and lamella (r = 0.0 cm) dried faster than the internal layers. Approximately at t = 15 h, the a_w_ of the outermost cap layer and the lamella decreased rapidly, while the center remains at initial aw until the drying front reaches the center after 40 h, near the end of drying.

#### 5.5.2 Product temperature and distribution

**Fig. 10:**
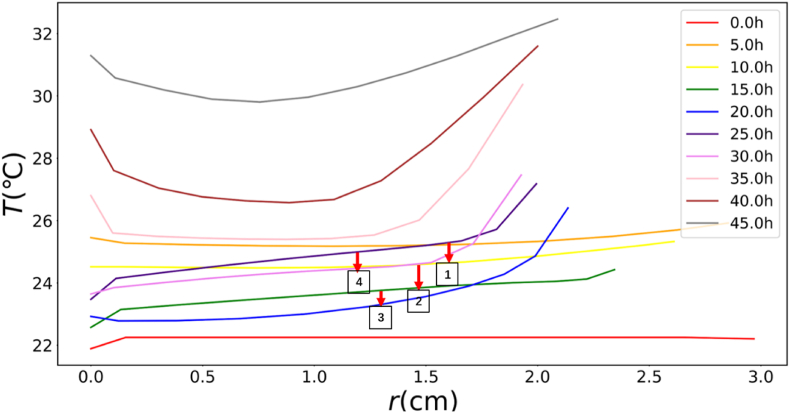
Product temperature distribution evolution at different time intervals during the drying (red arrows 1–4 indicate the change during the intervals of 5–10 h, 10–15 h, 15–20 h, and 25–30 h).

Fig. 10 depicts the model-derived temperature profiles showing changes in mushroom during drying. The initial drying stages exhibits flat and uniform profiles with minimal internal gradients. By 35 h, the surface and lamella temperatures exceeded those of the core, coinciding with their rapid aw reduction (Fig. 10). Diminished evaporative cooling in these drier regions allowed heat accumulation, while the still-moist core retained its cooling capacity. In late drying, temperature gradients diminished as the outer layers approached ambient temperature, eventually equilibrating the entire product to ambient temperature, coinciding with the aw distribution.

Furthermore, it is observed that the simulated center temperature decreases during the intervals of 5–10 h, 10–15 h, 15–20 h, and 25–30 h, as indicated by the red arrows in Fig. 10, when the temperatures were expected to increase. The plots showing the changes in center temperature during these time periods were enlarged and presented in Fig. S4 of the **Supplementary Materials (**Section 3.3**)**. These anomalous variations can be primarily attributed to fluctuations in relative humidity (RH), which intricately interact with the moisture dynamics within the mushroom.

Following the validation of the model's ability to predict the coupled evolution of moisture and temperature during drying and the clarification of its underlying mechanisms, the core innovations and advances of this model are summarized through a comparison with existing ones. Firstly, unlike previous studies (Tran et al., 2020; Zecchi et al., 2011; Zhu et al., 2021), which treat mushrooms as homogeneous porous media, this model explicitly divides mushrooms into two thermodynamic phases (cell wall/cytoplasm and vacuoles) separated by intact cell membranes validated via NMR measurements, and distinguished the mushroom into cap and lamellar parts. This distinction captures water partitioning between phases and local distribution in mushroom, which is critical for understanding the real moisture sorption and transport mechanism (Jin et al., 2014). Secondly, this model integrates cellular-level thermodynamics (via the Flory-Huggins theory based on composition), the effect of viscoelastic relaxation on moisture sorption/diffusion (via Maxwell Model and Flory-Rehner theory), and multiphase heat/mass transfer. It bridges microstructural features (cell membrane integrity, rheological property) with macroscopic drying behavior, addressing gaps in existing multiphase models that ignore biological complexity of cellular food materials (Onwude et al., 2021; Purlis, 2019; Zhu et al., 2021). Additionally, this model employs predictive properties, such as the mutual moisture diffusion coefficient and composition-weighted thermal conductivity, which adapt to temperature and moisture changes. This captures the evolving physical nature of mushroom tissue during drying, which is missing in simplified models (Tran et al., 2020; Zecchi et al., 2011). However, it must be noted the current model is missing a mechanistic model, describing the large deformation and wrinkling of the mushroom during drying.

### 5.6 Identification of cell membrane integrity change with NMR measurement

**Fig. 11:**
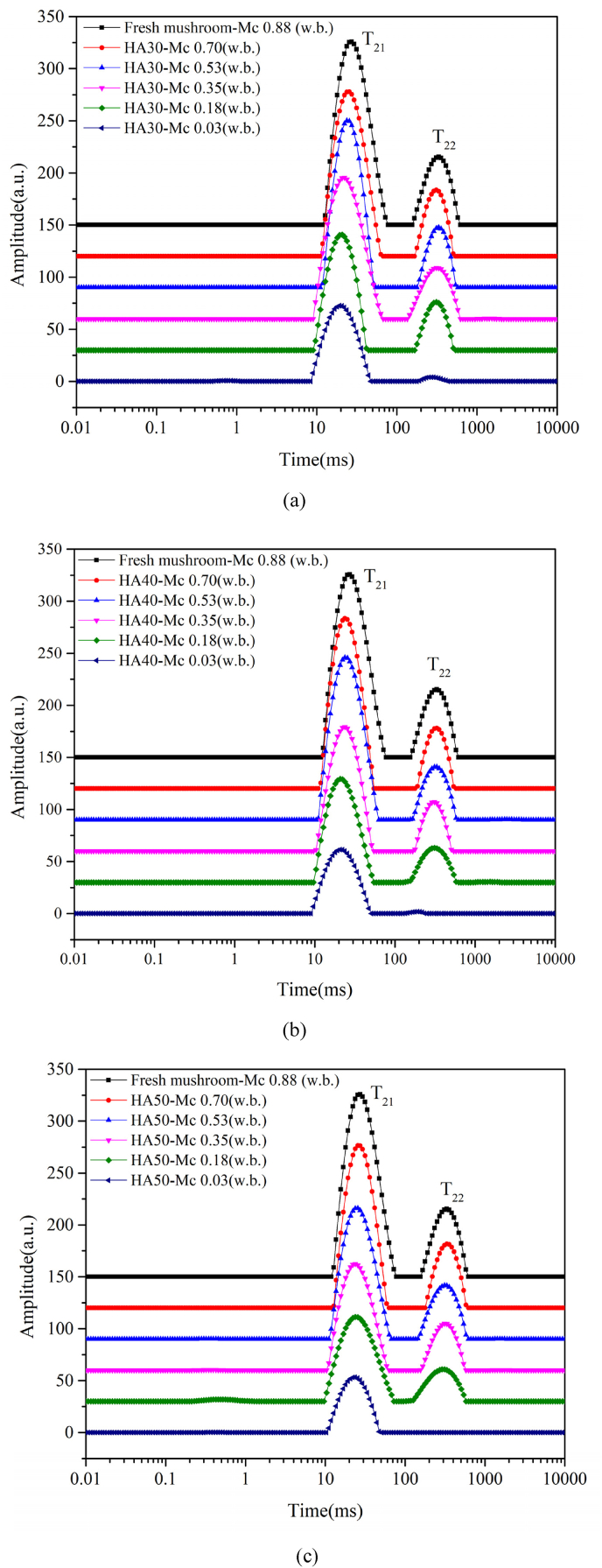
The reproduced T_2_ inversion spectra curves of fresh and dried mushrooms from (Qiu et al., 2021).

With NMR, two distinct thermodynamic phases in hyphae were identified, separated by the cell membrane and distinguishable by their respective T_2_ peaks (as detailed in Section 5.1). In an earlier study using the conductivity method, it showed that the loss of cell membrane integrity occurred in a critical temperature regime (T ~ 40 °C) (Hu et al., 2022). While the conductivity method is straightforward, it is an indirect measurement, and via T_2_-NMR, one obtains a direct, non-destructive indication of the cell membrane integrity via the number of water populations. Upon loss of cell membrane integrity, it is expected the T_21_ and T_22_ populations to merge into one. Here, the NMR results of mushroom during in-situ hot air drying at 30, 40, 50 °C in another study was reproduced (Qiu et al., 2021), as shown in Fig. 11. These results were compared with the data based on conductivity. However, during NMR measurement the product temperature was not recorded, whereas the moisture content was recorded. The validated drying model can be used for the prediction of product temperature. Based on the predicted product temperature and measured moisture content, the product state is known, and the NMR-results can be plotted (i.e. the presence of one or two water populations) in the previous contour plot based on conductivity (Hu et al., 2022). To objectively infer the presence of one or two peaks, a threshold of 5 % (relative to the peak of the fresh mushroom) was used, and the presence of peaks above this threshold was counted.

**Fig. 12:**
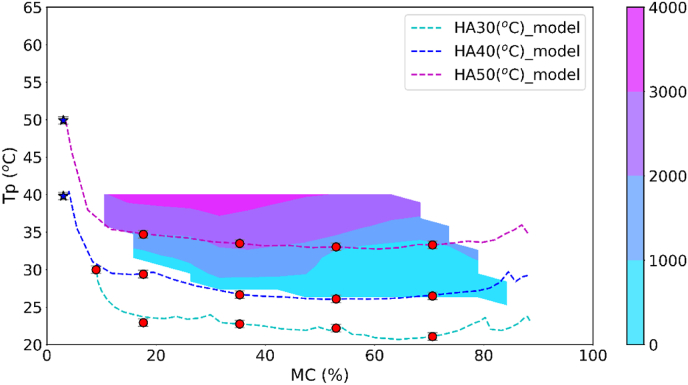
Model-simulated moisture–temperature state diagram of hot air-dried mushrooms with contour lines for the electrical conductivity (μS/cm) of rehydration liquid (The red circles denote samples with two T_2_ peaks, and blue stars represent those with one T_2_ peak.).

The evolution of the product state (T_p_ and y_w_), as predicted by the validated drying model, is plotted in the conductivity contour plot, together with symbols indicating the presence of one or two peaks, as shown in Fig. 12 The color bar indicates the values of conductivity. Lower conductivity indicates less cell membrane damage (Hu et al., 2022). From the contour plot, it is observed that two critical conditions for the loss of cell membrane integrity: T_p_ > 40 °C or y_w_<3 %. The first threshold is imposed by a (fluid-to-gel) phase transition of the lipid bilayer (Gonzalez et al., 2010; Singhal et al., 2020). This second condition is probably caused by osmotic stress. However, it is expected that under these conditions, the loss of cell membrane integrity is reversible, as shown in the previous study that mushrooms after HA35 drying treatment can restore turgor after rehydration.

## 6 Conclusion

This study presents a mechanistic multiphase and multiscale model for mushroom drying. This model reasonably captures the moisture content evolution and gradients variation, accounting for the thermal equilibrium between the two thermodynamic phases within the hypha via the Flory-Huggins theory, viscoelastic relaxation of the cell wall by Maxwell relaxation modes, and the heat and mass transfer mechanism accounting for the unique porous structure of mushroom filled with vapor and air. The analysis of experiments with the model shows the particular drying behavior of mushrooms: the inclusion of these factors enables the reasonable prediction of moisture and temperature evolution, such as the prolonged high central aw due to internal evaporation cooling. Furthermore, the model was used to predict the product temperature profile during hot air drying at 30 °C, 40 °C, 50 °C. Integration of these predictions with conductivity and NMR data allowed us to determine that preserving membrane integrity requires maintaining temperatures below 40 °C and moisture content above a threshold of 3 % (w.b.). As the study showed preservation of the cell membrane integrity is required for optimal rehydration of *shiitake* mushrooms, the moisture-temperature-conductivity contour plot integrated with T_2_ relaxation results indicating the cell membrane status provides a convenient tool for the optimization for critical drying conditions of serial combined drying processes of cellular food materials.

## CRediT authorship contribution statement

Lina Hu: Conceptualization, Investigation, Experimental Data collection, Formal analysis, Writing – original draft. Xin Jin: Supervision, Conceptualization, Writing – review & editing, Funding Acquisition. Yitong Xie: Experimental Data collection. Jinfeng Bi: Conceptualization, Supervision, Funding Acquisition. Ruud van der Sman: Supervision, Conceptualization, Writing – review & editing.

## Declaration of competing interest

One of the authors is guest editor of this special issue. The review process is handled by independent editor.
