# Supplementary material for: A multiphase and multiscale mechanistic model for hot air drying of shiitake mushroom
Source: Curr Res Food Sci. 2025 Dec 29;12:101296. doi: 10.1016/j.crfs.2025.101296 (PMC12861252; doi:10.1016/j.crfs.2025.101296)
Supplement: Multimedia component 1 [file mmc1.docx]

**Supplementary Material**

**1.Supplementary for modeling description**

**1.1 The main compounds composing *shiitake* mushroom**

The main compounds present in *shiitake* mushroom are listed in Table S1.

Table. S1 Mass fraction of components in dry matter of *shiitake* mushrooms

| Component  Location  Content | Mannitol | Trehalose | Fiber | Protein | Ash (KH_2_PO_4_) |
| --- | --- | --- | --- | --- | --- |
| Phase1 | √ | √ |  |  | √ |
| Phase2 |  |  | √ | √ |  |
|  | 0.307 | 0.033 | 0.375 | 0.17 | 0.0335 |

- 1. **The large deformation version of the Maxwell mode**

The classical Maxwell model is applicable to small deformations of viscoelastic media. For our discussion later, it is convenient to rewrite the Maxwell model in terms of the elastic strain $\epsilon_{el}$ and inelastic strain $\epsilon_{in}=\epsilon-\epsilon_{el}$. The inelastic strain can relax as:

$\frac{{d\epsilon}_{in}}{dt}=\frac{\epsilon-\epsilon_{in}}{\tau_{0}}$ （S1）

$\tau_{0}$ is the viscoelastic relaxation time.

The stress is only due to the elastic part, which means

$\boldsymbol{\sigma}=G\epsilon_{el}$ (S2)

$G$ is the elastic (shear) modulus.

In our case we have to deal with the large deformation of the cell wall, then we need to convert the Maxwell model. For large deformations one use stretch parameter $\lambda=1+ \epsilon$ to express the strain. To describe the viscoelasticity of large deformation, we apply multiplicative decomposition to $\lambda$ :

$\lambda= \lambda_{el}\lambda_{in}=\left( 1+\epsilon_{el} \right)\left( 1+\epsilon_{in} \right)$（S3）

with the elastic part $\lambda_{el}$ and the inelastic part $\lambda_{in}$, respectively. We can find this definition is consistent with the small deformation when expanding this equation, due to the $\epsilon_{el}\epsilon_{in}$ is very small and can be neglected.

We assume isotropic deformation of mushroom, then we can use the volume fraction to indicate the deformation as done in the Flory-Rehner theory. Hence, we can apply multiplicative decomposition to $\frac{\phi_{s}}{\phi_{dry}}$, as for plastic deformations.

$\frac{\phi_{s}}{\phi_{dry}}=\frac{\phi_{s}}{\phi_{ref}}\frac{\phi_{ref}}{\phi_{dry}}= \frac{1}{\lambda_{el}^{3}\lambda_{in}^{3}}$（S4）

$\varphi_{s}$ is the polymer volume fraction, and $\varphi_{ref}$ is a reference value. $\phi_{dry}=1$.

The stress is the function of elastic deformation

$\boldsymbol{\sigma}=G*\left( \tilde{\varphi}^{\frac{1}{3}}-\tilde{\varphi} \right)$ （S5）(Sman, 2023)

We reinterpret$\varphi_{ref}$ as the internal variable describing the viscoelastic state, as in case of transient networks models (Curatolo et al., 2018; Reese, 2003). Based on the transient network hypothesis, the distance between physical crosslinks along the polymer backbone changes due to the mechanical deformations, presenting the viscoelastic relaxation of polymers, which is represented by $\varphi_{ref}$, as follows from the c*-theorem from deGennes (van der Sman, 2015).

A single relaxation time viscoelastic process at large deformations can be captured via the following relaxation of $\varphi_{ref}$(Sman, 2023):

$\frac{{d\phi}_{ref}}{dt}=\frac{\phi_{s}-\phi_{ref}}{\tau_{0}}$ （S6）

whose physical meaning is that the intermediate (stress free) reference state of polymer networks tends to relax towards the current state, as represented by $\phi_{s}$. The process is schematically shown in Fig.2.


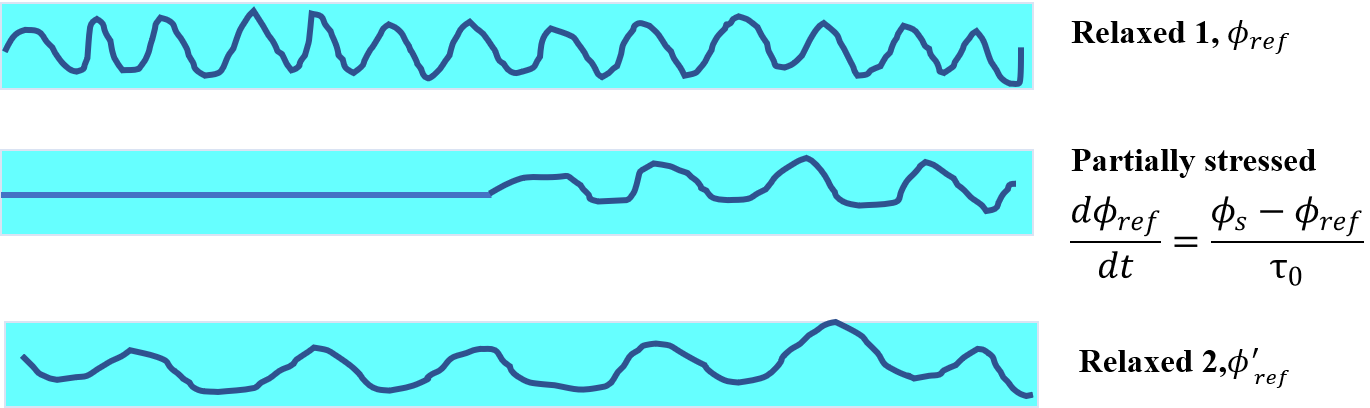


Fig. S1. Volume fraction of viscoelastic polymer changes with time

**1.3** **Material properties**

The constant parameters used to solve the mathematical model in Table S2.

**Table S2**

**Values of parameters present in the model**

|  | **Parameter** | **Value** | **Unit** | **Reference** |
| --- | --- | --- | --- | --- |
| $\rho_{water}$ | Density of water | 998 | kg/m ^3^ | (Çengel and Boles, 2006) |
| $\rho_{solid}$ | Density of solid matrix | 1591 | kg/m | (Oikonomopoulou and Krokida, 2012) |
| $\rho_{carb}$ | Density of carbohydrate | 1550 | kg/m ^3^ | (van der Sman, 2008) |
| $\rho_{pro}$ | Density of protein | 1330 | kg/m ^3^ | (van der Sman, 2008) |
| $\rho_{ash}$ | Density of ash | 2440 | kg/m ^3^ | (van der Sman, 2008) |
| $\rho_{fat}$ | Density of fat | 930 | kg/m ^3^ | (van der Sman, 2008) |
| $ƞ_{f}$ | Effective viscosity of mannitol | 1.00× 10 ^-3^ |  | (van der Sman and Meinders, 2013) |
| $K_{ss}$ | Free volume parameter | 69.21 | K | (van der Sman and Meinders, 2013) |
| $K_{ws}$ | Free volume parameter | 3.36× 10 ^-4^ | K |  |
| $K_{sw}$ | Free volume parameter | -19.73 | K | (van der Sman and Meinders, 2013) |
| $K_{ww}$ | Free volume parameter for water | 1.95× 10 ^-3^ | K | (van der Sman and Meinders, 2013) |
| $K_{ss}$ | Free volume parameter | 69.21 | K | (van der Sman and Meinders, 2013) |
| $T_{gs}$ | Glass translation temperature of solid | 347.15 | K | (Zhu et al., 2021) |
| $T_{gw}$ | Glass temperature of water | 136 | K | (van der Sman and Meinders, 2013) |
| $r_{H}$ | Hydrodynamic radius of mannitol molecule | 0.448× 10 ^-9^ |  | (van der Sman and Meinders, 2013) |
| $h_{evap}$ | Latent heat of evaporation | 2.26 × 10 ^6^ | KJ/kg | (Zhu et al., 2021) |
| $\delta$ | Shape factor | 0.79 |  |  |
| $V_{cs}$ | Specific free volume fraction for solute | 0.91 |  | (van der Sman and Meinders, 2013) |
| $V_{cw}$ | Specific free volume fraction for water | 0.59 |  | (van der Sman and Meinders, 2013) |
| $c_{p,solid}$ | Specific heat capacity of solid matrix | 1500 | J/kg/K | (Tansakul and Lumyong, 2008) |
| $c_{p,w}$ | Specific heat capacity of water | 4182 | J/kg/K | (Rakesh et al., 2012) |
| $c_{p,v}$ | Specific heat capacity of vapor | 2062 | J/kg/K | (Rakesh et al., 2012) |
| $c_{p,air}$ | Specific heat capacity of air | 1006 | J/kg/K | (Rakesh et al., 2012) |
| $\lambda_{air}$ | Thermal conductivity of air | 0.026 | W/m/K | (Rakesh et al., 2012) |
| $\lambda_{carb}$ | Thermal conductivity of carbohydrate | 0.2 | W/m/K | (van der Sman, 2008) |
| $\lambda_{pro}$ | Thermal conductivity of protein | 0.18 | W/m/K | (van der Sman, 2008) |
| $\lambda_{ash}$ | Thermal conductivity of ash | 0.33 | W/m/K | (van der Sman, 2008) |
| $\lambda_{fat}$ | Thermal conductivity of fat | 0.18 | W/m/K | (van der Sman, 2008) |
| $\lambda_{water}$ | Thermal conductivity of water | 0.599 | W/m/K | (Rakesh et al., 2012) |

***Moisture diffusion coefficient*** $\boldsymbol{D}_{\boldsymbol{w}_{\boldsymbol{diff}}}$

To make the model as mechanistical as possible, we assume the moisture diffusion coefficient $\boldsymbol{D}_{\boldsymbol{w}_{\boldsymbol{diff}}}$ as a mutual diffusion coefficient, which is based on three theories: the free volume theory for the water self diffusivity; the generalized Stokes–Einstein relation for the solute self diffusivity and the Darken relation linking the mutual diffusivity to the self diffusivities of water and solutes (van der Sman and Meinders, 2013).

$D_{w_{diff}}= \phi_{w}*D_{s, s}+\left( 1- \phi_{w} \right)*D_{s,w}$ (S7)

$D_{s, s}$ and $D_{s,w}$ are the self diffusivities of the solute and water respectively. During drying, the moisture within the mushroom is unevenly distributed. In the low moisture regime, diffusion is dominated by the water self diffusivity $D_{s,w}$, while in the high moisture regime, it is governed by the solute self diffusivity $D_{s, s}$ However, a major part of the dry matter of mushrooms consists of biopolymers located in cell wall and cell cytoplasm which do not include the generalized Stokes–Einstein as solutes. Hence, to account for these deviations from theory, we multiply the diffusivity with a pre-factor $F_{w,diff}$ as shown in Eq.S7.

We approximate the solute self diffusivity with a variable simplification by taking the molecule of mannitol, which is the main component in mushroom dry matter, to describe the hydrodynamic radius r_H_.

$D_{selfdiffusion solute}=\frac{k_{B}T}{6\piƞ_{f}r_{H}}$ (S8)

With $k_{B}$ the Boltzmann constant (1.380649 × 10^-23^ J/K), T the product temperature, $r_{H}$ the hydrodynamic radius of the mannitol molecule, and $ƞ_{f}$ the effective viscosity of the mannitol.

$D_{selfdiffusion water}=D_{0}*e^{\frac{-\Delta E}{RT}}*e^{\frac{-y_{w}*V_{cw}+\delta{*y_{s}*V}_{cs}}{y_{w}*K_{ww}*\left( K_{sw}-T_{gw}+T_{p} \right)+y_{s}*K_{ws}*\left( K_{ss}-T_{gs}+T_{p} \right)}}$ (S9)

With $\Delta E$ the activation energy, R universal gas constant (8.314 J/mol/K) $K_{ij}$ free volume parameters, $\delta$ is a shape factor. $y_{w}$ and $y_{s}$ are the mass fraction of water and solutes, respectively, and $V_{cw}$ and $V_{cs}$ are related to the specific free volume fraction for water and solute respectively. These parameter values are obtained from (van der Sman and Meinders, 2013), which are listed in Table S2.

***Air/vapor diffusion coefficient***

The air/vapor diffusion coefficient in gas within the control volumes, $D_{{gas}_{air}}$ and $D_{{gas}_{vap}}$, are multicomponent Fick diffusivities (Bird et al., 2002), and can be written as:

$D_{{gas}_{vap}}=0.129e^{-4}+0.18e^{-6}\left( T-T_{0} \right)$ (S10)

***Thermal parameters***

The physical thermal parameters of mushroom are based on composition and effective medium theory. In porous materials, such as mushroom containing air (gas phase) dispersed in the hypha matrix (composed of water and solids), the thermal conductivity λ is determined by the complex interplay of hypha and gas phases. According to the Maxwell-Eucken model, the dispersed phase (air in the mushroom) is modeled as spherical particles uniformly distributed in the continuous phase (hypha matrix of the mushroom).

The thermal conductivity $\lambda_{mushroom,eff}$ and specific heat capacity $c_{p,mushroom}$ (J/kg/K) are calculated as follows:

$\lambda_{mushroom,eff}=\lambda_{hypha}\frac{1+\left( \varphi_{air}+\varphi_{hypha}*Q \right)*\zeta}{\left( 1+\varphi_{hypha}*Q*\delta\right)}$ (S11)

$\lambda_{mushroom,lam,eff}=\varphi_{lamella}\lambda_{mushroom,eff}+\left( 1-\varphi_{lamella} \right)\lambda_{air}$ (S12)

$Q=\frac{1}{3}$ (isotropic), ζ$=\left( \lambda_{\mathrm{air}}-\lambda_{\mathrm{hypha}} \right)/\lambda_{\mathrm{hypha}}$,

$\lambda_{\mathrm{hypha}}=\left（ \varphi_{s}\lambda_{\mathrm{solid}}+\varphi_{w}\lambda_{water} \right）/(1-\varphi_{air})$ (S13)

$$\lambda_{\mathrm{solid}}=\varphi_{carb}\lambda_{carb}+\varphi_{pro}\lambda_{pro}+\varphi_{ash}\lambda_{ash}+\varphi_{fat}\lambda_{fat}$$

$c_{p,mushroom}=\frac{{\varphi_{w}c}_{p,water}\rho_{water}+{\varphi_{s}c}_{p,solid}\rho_{solid}}{\rho_{mushroom}}$ (S14)

Here,

$\rho_{mushroom}=\rho_{water}\varphi_{w}\boldsymbol{+}\rho_{solid}\varphi_{s}+\rho_{air}\varphi_{air}$ (S15)

With $\rho_{solid}=\frac{\sum X_{i}}{\sum\frac{X_{i}}{\rho_{i}}}$ ; $c_{p,solid}=\sum\rho_{i}Ф_{i}$ ; $Ф_{i}=\frac{X_{i}}{\rho_{i}\sum\frac{X_{i}}{\rho_{i}}}$

$X_{i}$ is the mass fraction based on the total dry matter, as listed in Table1, and $Ф_{i}$ represents the volume fraction. $i$ indicates carbohydrate, protein, ash, and fat, respectively. Note, we assume that the contribution of vapor (gas) to the specific heat capacity of mushroom is negligible because of its low density.

***Viscoelastic property of mushroom***

From the compression measurement on mushrooms with the moisture contents ranging from 0.20-6.46 g/g dry basis (d.b.). dried at 35 ℃ at the tissue scale (Hu et al., 2023), we established the moisture-dependency functions for the elastic modules $G_{0}$ and relaxation time $\tau_{0}$. Note, the values of $G_{0}$ for the cell wall of mushroom are assumed to be higher than those at the tissue scale. This is because of the existence of stiff chitin fibers in the cell wall, and at the tissue scale, easy sliding of hyphae leads to a moderately effective G (Ene et al., 2015). Therefore, in this study these functions are applied to mushroom samples, and multiplied with unknown pre-factors $F_{G_{i}}$ and $F_{{\tau0}_{i}}$ which need to be fitted. Here, $i$ indicates the slow and fast viscoelastic relaxation modes as mentioned above. Therefore, the generalized functions between the modules/relaxation time and moisture content ($y_{w}$ $d.b.$) of on the powder scale can be expressed as follows:

when $y_{w}>0.91$,

$G_{0}=F_{G_{i}}*(42.85+161.26*\left( \frac{y_{w}-0.91}{5.54} \right))$ (S16)

$\tau_{0}=F_{{\tau0}_{i}}*\left( 2.17+1.19*\left( \frac{y_{w}-0.91}{5.54} \right) \right)$ (S17)

when $y_{w}<0.91$,

$G_{0}=F_{G_{i}}*(42.85+872.55*\left( \frac{y_{w}-0.91}{-0.7} \right))$ (S18)

$\tau_{0}=F_{{\tau0}_{i}}* (2.17-0.72*\left( \frac{y_{w}-0.91}{5.54} \right))$ (S19)

**1.4 Numerical implementation**

Finite Volume Mass equations for liquid water, gas and water vapor (Eqs.5--14) were solved by finite volume analysis, central differencing and Euler forward. The total simulation time is set to be consistent with the end time of drying experiments, and the time step is set based on the heat transfer penetration on the surface and lamellar layer of the mushroom. The Fourier number ${Fo}^{*}$ is defined as the ratio of the rate of heat conduction to the rate of heat storage. For an explicit scheme to be stable in a one-dimensional heat conduction problem, the Grid Fourier number must satisfy the condition ${Fo}^{*}\leq0.2$. We take the minimum value of the multiplication of the Grid Fourier number (${Fo}^{*}=0.2$) and thermal capacity and resistance as the time step. The mesh is applied to discretize computational domain. As shown in Fig.2, the cap mushroom is divided into hemispherical shells as the control volumes with the equal thickness $\triangle r=\frac{Radius}{10}$ .The lamella part is a single control volume, which connects all hemispherical shells at its top, and the environment at its bottom surface. Python was used to solve the discretized governing equations. The shrinkage of *shiitake* mushroom results in changes in diameter of the mushroom and the $\triangle r$ of control volumes, which are recalculated and updated at every time step according to the changes in the volume of water and gas during drying.

**1.5 Parameter fitting results for hot air drying model**

Single factor tests were conducted on parameters $e$, $F_{w,conv}$ and $F_{w,diff}$, respectively. The parameter range and values of the objective function are listed in Table 2. The initial guess of these parameters are as follows: $e$ ranges from 0.1 to 0.9, $F_{w,conv}$ ranges from 1 to 20, $F_{w,diff}$ ranges from 1 to 20. From the single-factor tests, we obtained the optimal parameter set, as shown in test 10.

Table S2. Single-factor tests

| Test code | $e$ | $F_{w,conv}$ | $F_{w,diff}$ | $LSE\_sum$ |
| --- | --- | --- | --- | --- |
| 1 | 0.1 | 5 | 5 | 1.181 |
| 2 | 0.5 | 5 | 5 | 0.996 |
| 3 | 0.9 | 5 | 5 | 0.832 |
| 4 | 0.9 | 1 | 5 | 1.088 |
| 5 | 0.9 | 5 | 5 | 0.832 |
| 6 | 0.9 | 10 | 5 | 0.814 |
| 7 | 0.9 | 20 | 5 | 1.544 |
| 8 | 0.9 | 10 | 1 | 2.196 |
| 9 | 0.9 | 10 | 5 | 0.814 |
| 10 | 0.9 | 10 | 10 | 0.765 |
| 11 | 0.9 | 10 | 20 | 0.949 |

Subsequently, multiple rounds of parameter refinement were performed to further optimize the identified optimal parameter set. As shown in Table S3, to explore the local parameter space around the initial optimal values, the first round (tests 12-16) refined values were selected for each parameter: for $e$, values of 0.5 and 0.7 were added to verify if 0.9 was indeed the optimal maximum; for $F_{w,conv}$, values of 5 and 15 were tested, bracketing the initially optimal value of 10; for $F_{w,diff}$, 5 and 15 were examined. The results of this round revealed a new optimal combination (test 14): $e$ = 0.9, $F_{w,conv}$ = 5, and $F_{w,diff}$ = 10, which improved the response variable compared with the previous optimal.

Consequently, in test 17-20, we continued the parameter refinement for the second round to further explored the local parameter space, and revealed a new optimal combination (test 19): $e$ = 0.9, $F_{w,conv}$ = 5, and $F_{w,diff}$ = 8, with a response value of 0.7418249 (representing a 2.3% improvement over the previous optimal value of 0.759156). In test 21-24, a third round of refinement was designed to further explore potential improvements. A focused set of experiments was conducted to investigate the effects of adjusting the parameters within narrower intervals around the newly identified optimal values. To determine whether values of $F_{w,diff}$ smaller than 8 could yield further improvements, two additional levels 6,7 were tested. Given that $F_{w,conv}$ = 5 was optimal in the first-round tests (among 4, 5, and 6), smaller values of 3 was explored to check for potential hidden minima. The combination of $F_{w,conv}$ = 3 and $F_{w,diff}$ =7 was selected to evaluate the interaction between the smaller $F_{w,conv}$ and adjusted $F_{w,diff}$ values.

The results showed that despite testing multiple combinations, the new optimal response value of 0.745802 (corresponding to $e$ = 0.9, $F_{w,conv}$ = 5, $F_{w,diff}$= 7) was higher than the previous optimum. This indicates that reducing $F_{w,conv}$or $F_{w,diff}$ within the current parameter refinement did not improve the outcome; instead, it led to a worse response. Therefore, after multiple rounds of parameter refinement, the combination of $e$ = 0.9, $F_{w,conv}$ = 5 and $F_{w,diff}$= 8 remains the optimal solution.

Table S3. Parameter Refinement tests

| Test code | $e$ | $F_{w,conv}$ | $F_{w,diff}$ | $LSE\_sum$ |
| --- | --- | --- | --- | --- |
| 12 | 0.5 | 10 | 10 | 0.799 |
| 13 | 0.7 | 10 | 10 | 0.773 |
| 14 | 0.9 | 5 | 10 | 0.759 |
| 15 | 0.9 | 15 | 10 | 0.906 |
| 16 | 0.9 | 10 | 15 | 0.857 |
| 17 | 0.9 | 4 | 10 | 0.756 |
| 18 | 0.9 | 6 | 10 | 0.761 |
| 19 | 0.9 | 5 | 8 | 0.742 |
| 20 | 0.9 | 5 | 12 | 0.790 |
| 21 | 0.9 | 5 | 7 | 0.746 |
| 22 | 0.9 | 5 | 6 | 0.771 |
| 23 | 0.9 | 3 | 8 | 0.750 |
| 24 | 0.9 | 3 | 7 | 0.762 |

**1.6 The calculation and meanings of the statistical parameters**

Coefficient of Determination (R²), assesses the goodness-of-fit. It is given by:

$R^{2}=1-\frac{\sum\left( y_{exp}-y_{pred} \right)^{2}}{{\sum\left( y_{exp}-\bar{y}_{pred} \right)}^{2}}$ (S20)

where $y_{exp}$ represents the experimental values, $y_{pred}$ are the predicted values, and $\bar{y}_{pred}$ is the mean of the experimental values.

Root Mean Square Error (RMSE), which measures the differences between the predicted and observed values:

$RMSE=\sqrt{\frac{\sum\left( y_{exp}-\bar{y}_{pred} \right)^{2}}{N}}$ (S21)

where 𝑛 is the number of observations.

Mean Absolute Error (MAE), which provides the average of absolute errors between the predicted and experimental values:

$MAE=\frac{\sum\left| y_{exp}-y_{pred} \right|}{N}$ (S22)

**2. Material and methods**

**2.1 Material preparation**

Fresh *shiitake* mushrooms were purchased from a mushroom farm in Beijing (China) and stored in plastic bags at 4 ℃ until further use. The mushroom stems were cut, leaving mushroom caps with a diameter of 60 ×10⁻³ m for experiments. The thickness of lamella layer of mushroom cap is about 2×10⁻³ m. The average moisture content of mushrooms was 89.14 ± 2.0% (wet basis: w.b.), which was measured in a drying oven (DHG-9123A, Shanghai Jinghong Experiment Facility Co., Ltd., Shanghai, China) at 105 ◦C for 24 h (AOAC, 1990).

**2.2** **Moisture content and temperature monitoring during drying**

The moisture content and temperature of the mushroom were measured during hot air drying at 35 ◦C (HA35), with an air velocity maintained at 2 m/s. A precisely measured quantity of fresh shiitake mushrooms (200.0 ± 1.0 g) was placed and evenly spread on a wire mesh tray. The moisture contents of mushrooms were measured after various drying durations: 6h, 12h, 24h, 36h, 48h. The surface (measured at a depth of 0.2 mm depth below the skin of the mushroom cap) and center (measured at a depth of 10 mm) temperatures of mushrooms were recorded using a K-type thermocouple probe (Omega Engineering Inc., Stamford, Connecticut, USA) connected to a data recorder (PicoLogTC-08, Pico Technology, England) (Hu et al., 2022).The air temperature and relative humidity within the drying oven were monitored synchronously using a humidity and temperature data logger (EL-USB-2-LCD+, Lascar Electronics, Hong Kong, China) during each experiment. These measurements were taken performed consecutively for each drying duration. The monitored air temperature and relative humidity results are given below, which provide the real information about the drying environment.


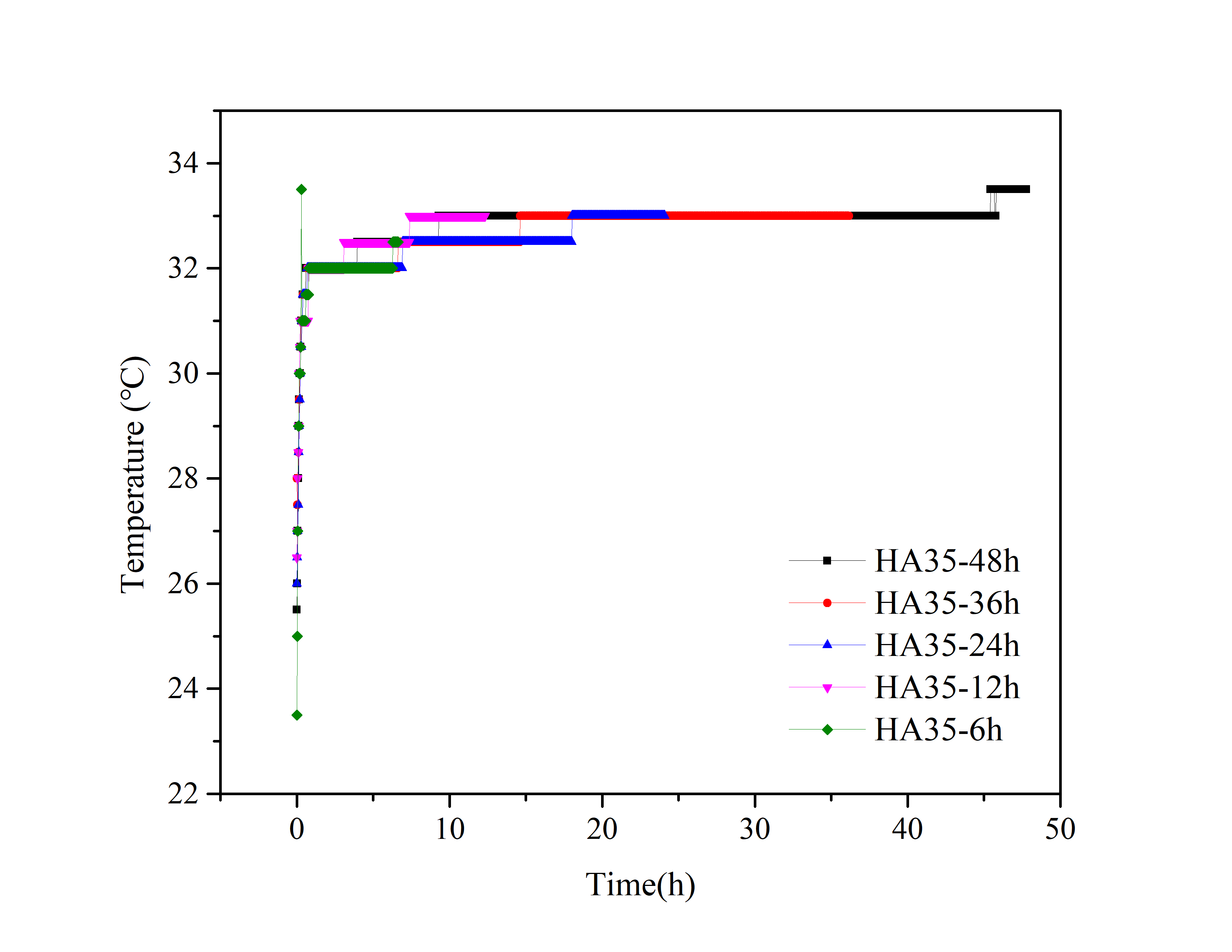


(a)


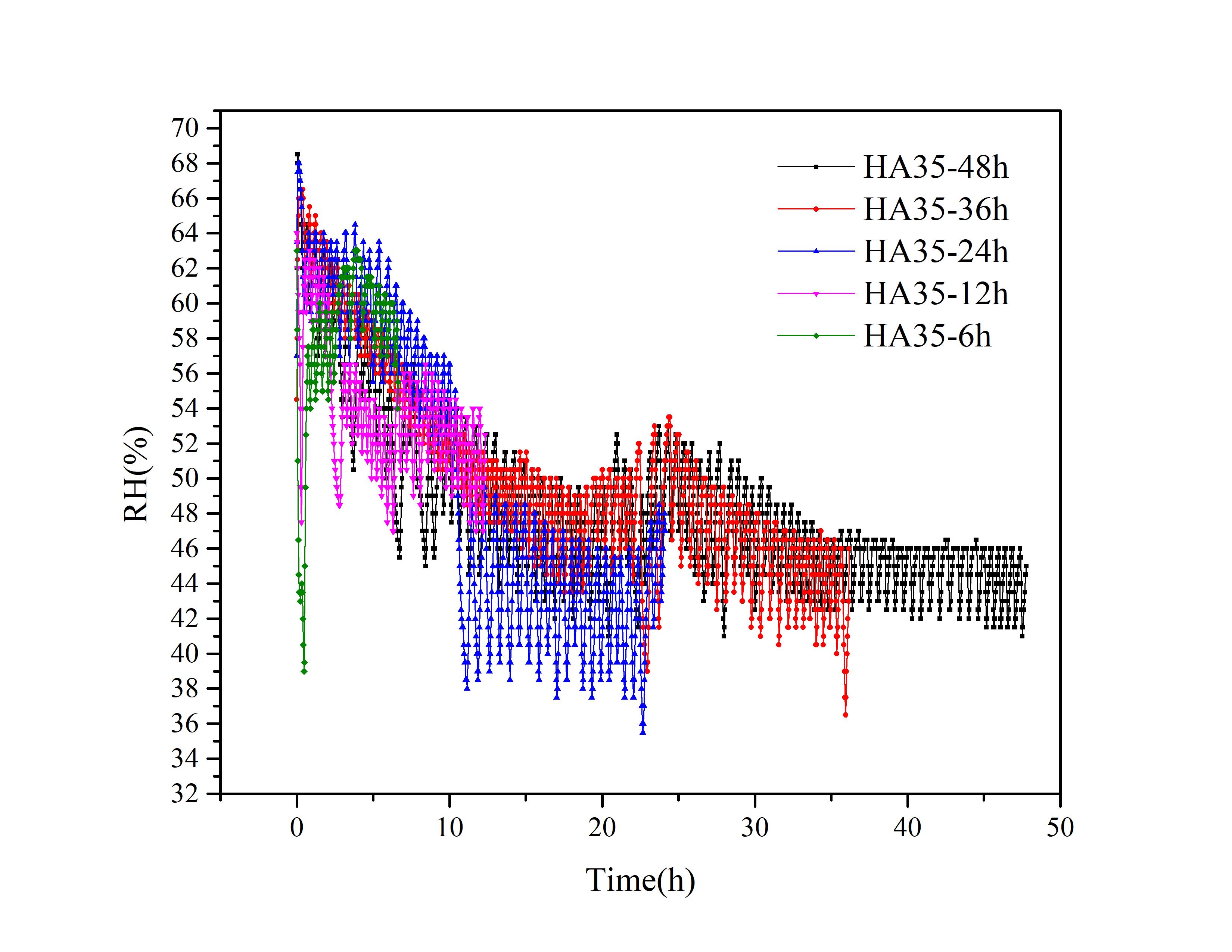


(b)

Fig. S2. The air temperature (a) and relative humidity (b) in the drying oven

**2.3 Measurement of** **dynamic moisture sorption of mushroom**

The dynamic moisture sorption (DVS) measurements were conducted on HA35 dried mushroom samples. The sample preparation and DVS measurement procedure are the same as those used in our previous study (Hu et al., 2023).

1. **Supplementary for results**

**3.1 The derivation procedure for the porosity evolution of mushrooms during drying**

$$V_{mushroom}\left( t \right)=V_{air}\left( t \right)+V_{solid}+V_{water}$$

$$\frac{V_{mushroom}\left( t \right)}{V_{solid}}=\frac{V_{air}\left( t \right)}{V_{solid}}+1+\frac{V_{water}\left( t \right)}{V_{solid}}$$

$$\frac{V_{mushroom}\left( t \right)}{V_{solid}}=\frac{V_{air}\left( t \right)}{V_{solid}}+1+\frac{\frac{m_{water}(t)}{\rho_{water}}}{\frac{m_{solid}}{\rho_{solid}}}$$

$$\frac{V_{mushroom}\left( t \right)}{V_{solid}}=\frac{V_{air}\left( t \right)}{V_{solid}}+1+\mathrm{Xm}\left( t \right)*\frac{\rho_{solid}}{\rho_{water}}$$

$$\frac{V_{mushroom}\left( t \right)}{V_{solid}}=\frac{V_{air}\left( t \right)}{V_{solid}}+1+\mathrm{Xm}\left( t \right)*\frac{\rho_{solid}}{\rho_{water}}$$

**3.2 Absolute humidity changes in the drying oven**


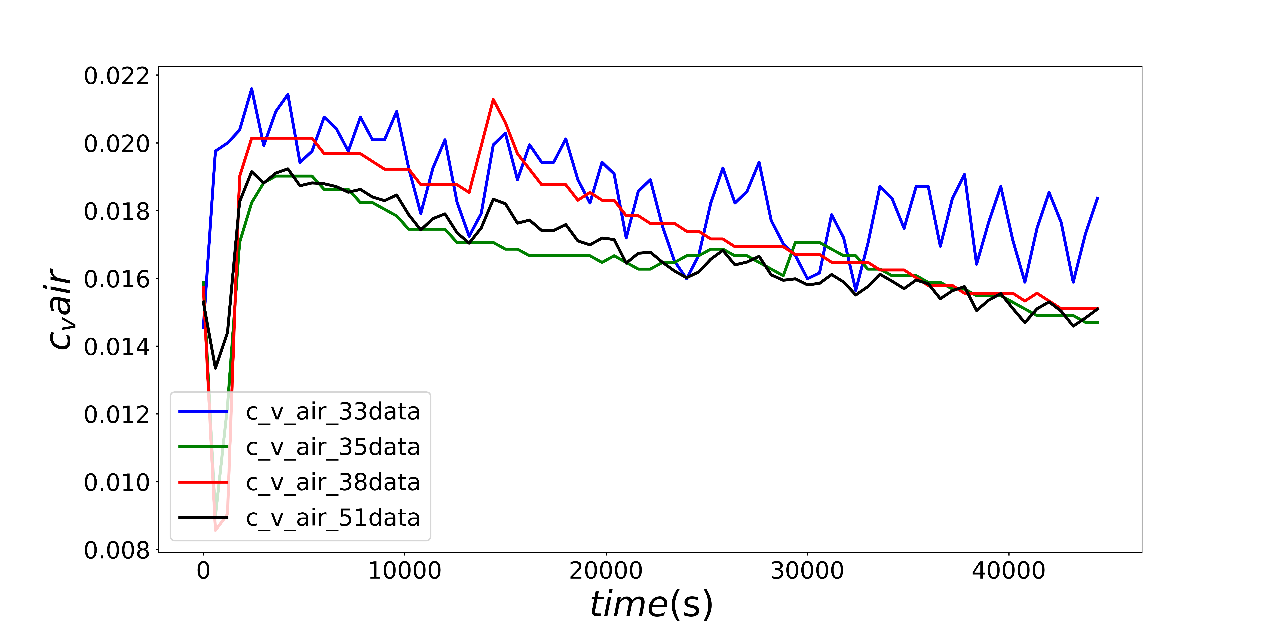
Fig.S3. Absolute humidity change in the drying oven during HA33, HA35, HA38, HA51

**3.3 Product temperature fluctuations during drying**


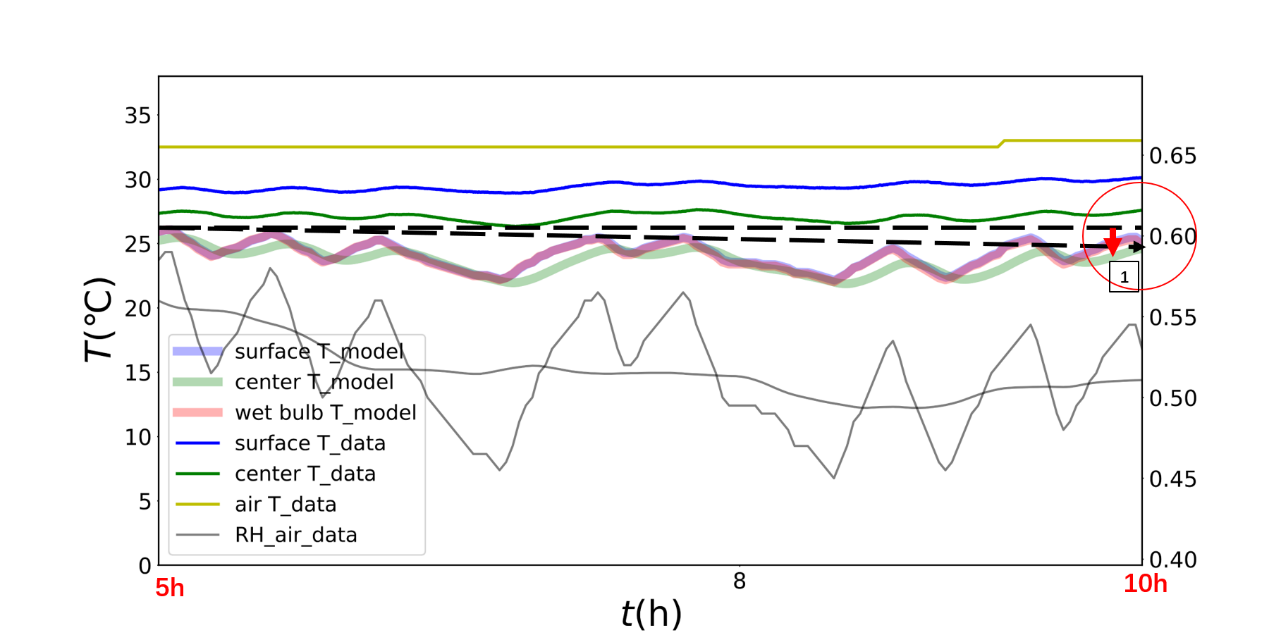


(a)


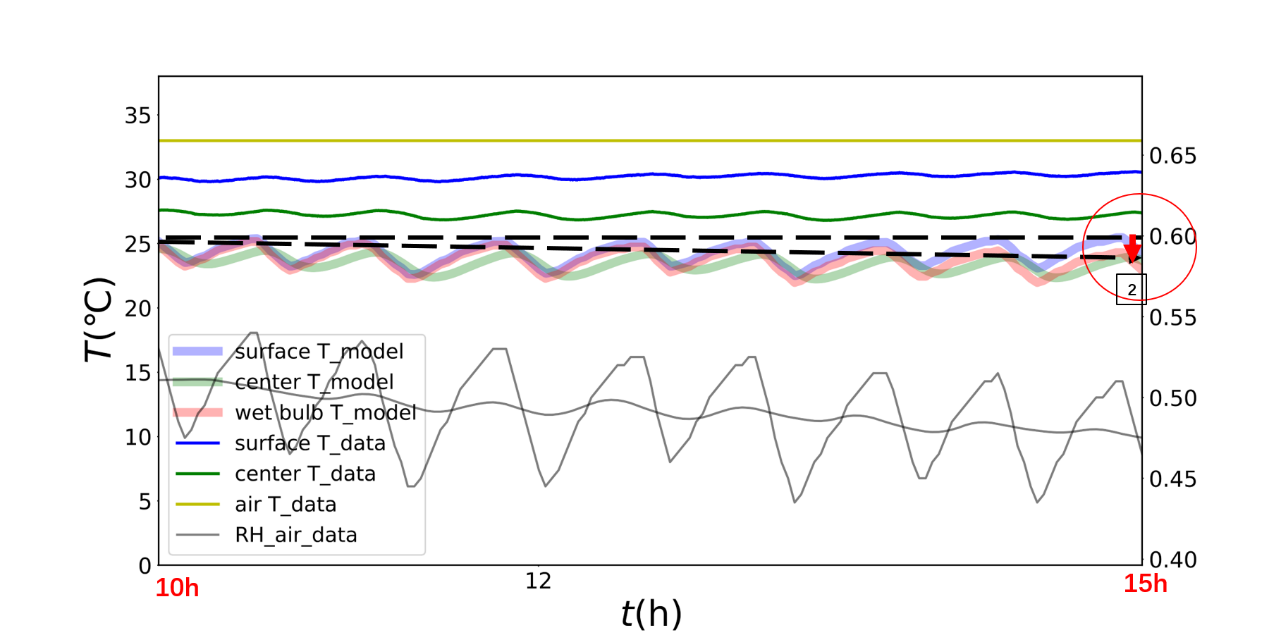


(b)


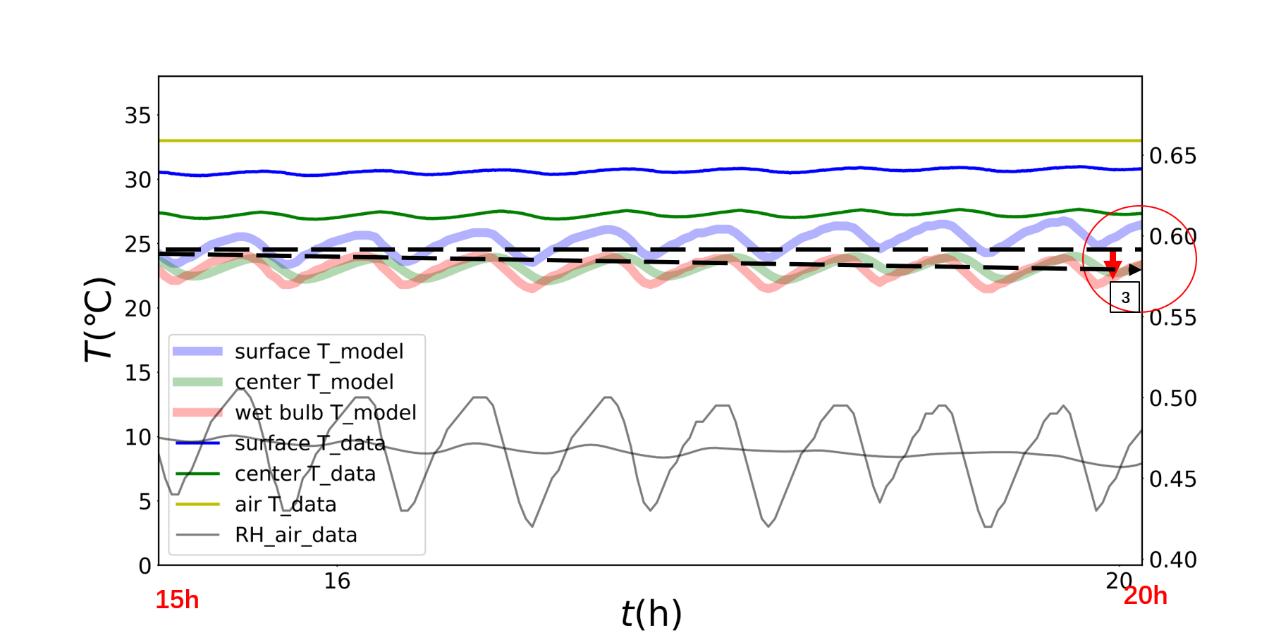


(c)


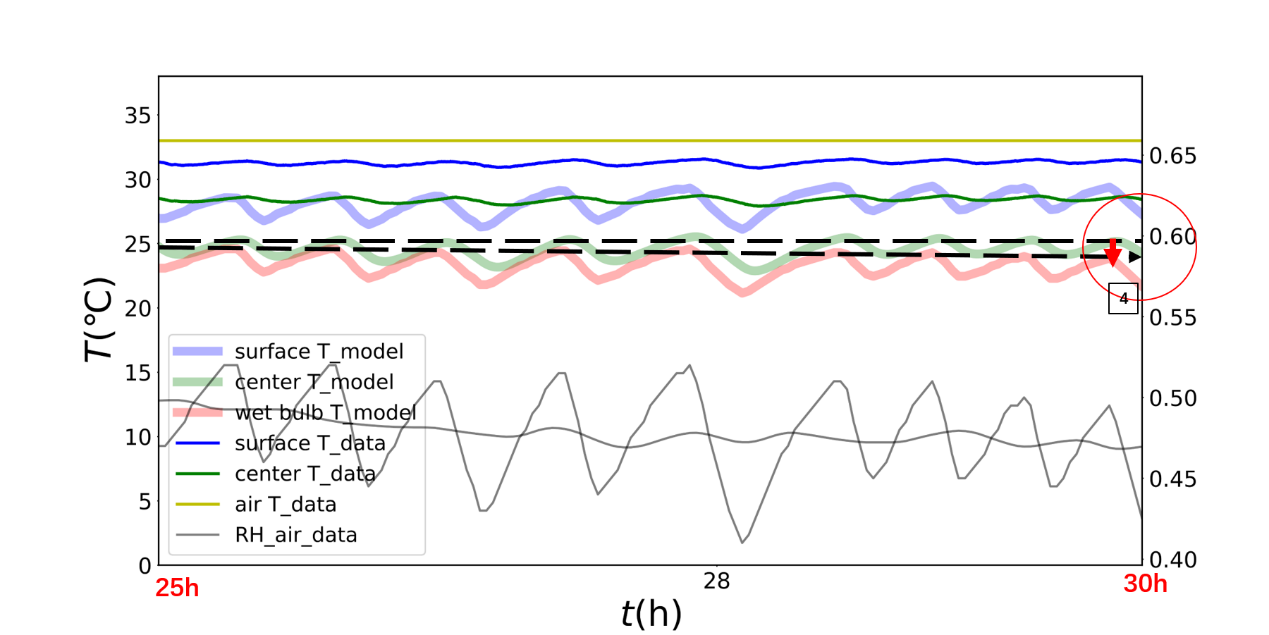


(d)

Fig. S4. Product temperature fluctuations align with relative humidity at different time intervals during the drying (red arrows 1-4 indicate the decrease during the intervals of 5 - 10 h(a), 10 - 15 h(b), 15 - 20 h(c), and 25 - 30 h(d))

**References**

Bird, R.B., Stewart, W.E., & Lightfoot, E.N. ( 2002). *Transport Phenomena (2nd Ed)_* (2nd Ed ed. John Wiley & Sons, Inc., New York / Chichester / Weinheim / Brisbane / Singapore / Toronto.

Çengel, Y., & Boles, M. (2006). *Thermodynamics: An Engineering Approach*. McGraw Hill Higher Education.

Curatolo, M., Nardinocchi, P., & Teresi, L. (2018). Driving water cavitation in a hydrogel cavity. *Soft Matter.* 14, 2310. <https://doi.org/> 10.1039/C8SM00100F.

Ene, I.V., Walker, L.A., Schiavone, M., Lee, K.K., Martin-Yken, H., Dague, E., Gow, N.A.R., Munro, C.A., & Brown, A.J.P. (2015). Cell Wall Remodeling Enzymes Modulate Fungal Cell Wall Elasticity and Osmotic Stress Resistance. *mBio.* 6(4), e00986-00915. <https://doi.org/> 10.1128/mBio.00986-15.

Hu, L., Bi, J., Jin, X., & Sman, R.v.d. (2022). Microstructure evolution affecting the rehydration of dried mushrooms during instant controlled pressure drop combined hot air drying (DIC-HA). *Innovative Food Science and Emerging Technologies.* 79 103056. <https://doi.org/> 10.1016/j.ifset.2022.103056.

Hu, L., Bi, J., Jin, X., & Sman, R.v.d. (2023). Impact of physical changes in mushroom on variation in moisture sorption. *Journal of Food Engineering.*351, 111506. <https://doi.org/> 10.1016/j.jfoodeng.2023.111506.

Oikonomopoulou, V.P., & Krokida, M.K. (2012). Structural Properties of Dried Potatoes, Mushrooms, and Strawberries as a Function of Freeze-Drying Pressure. *Drying Technology.* 30(4), 351–361.<https://doi.org/10.1080/07373937.2011.639475>.

Rakesh, V., Datta, A.K., Walton, J.H., McCarthy, K.L., & McCarthy, M.J. (2012). Microwave combination heating: Coupled electromagnetics- multiphase porous media modeling and MRI experimentation. *AIChE Journal.* 58(4), 1262–1278.

Reese, S. (2003). A micromechanically motivated material model for the thermo-viscoelastic material behaviour of rubber-like polymers. *International Journal of Plasticity.* 19, 909–940.

Sman, R.G.M.v.d. (2023). Effects of viscoelasticity on moisture sorption of maltodextrins. *Food Hydrocolloids.* 139, 108481. <https://doi.org/> 10.1016/j.foodhyd.2023.108481.

Tansakul, A., & Lumyong, R. (2008). Thermal properties of straw mushroom,. *Journal of Food Engineering,* 87(1), 91-98. <https://doi.org/> <https://doi.org/10.1016/j.jfoodeng.2007.11.016>.

van der Sman, R.G., & Meinders, M.B. (2013). Moisture diffusivity in food materials. *Food Chemistray* 138(2-3), 1265-1274. <https://doi.org/> 10.1016/j.foodchem.2012.10.062.

van der Sman, R.G.M. (2008). Prediction of enthalpy and thermal conductivity of frozen meat and fish products from composition data. *Journal of Food Engineering,* 84(3), 400-412. <https://doi.org/> 10.1016/j.jfoodeng.2007.05.034.

van der Sman, R.G.M. (2015). Biopolymer gel swelling analysed with scaling laws and Flory–Rehner theory. *Food Hydrocolloids.* 48, 94-101. <https://doi.org/> 10.1016/j.foodhyd.2015.01.025.

Zhu, Y., Wang, P., Sun, D., Qu, Z., & Yu, B. (2021). Multiphase porous media model with thermo-hydro and mechanical bidirectional coupling for food convective drying. *International Journal of Heat and Mass Transfer*. 175,121356 <https://doi.org/> 10.1016/j.ijheatmasstransfer.2021.121356.
